# The impact of the pandemic on non-COVID-19 causes of death in the United States: a multiple cause of death analysis

**DOI:** 10.1007/s10654-025-01214-z

**Published:** 2025-03-19

**Authors:** Yu Li, Hang Li, Tim Adair

**Affiliations:** Melbourne School of Population and Global Health, Nossal Institute for Global Health, The University of Melbourne, Level 2, 32 Lincoln Square North, Melbourne, VIC 3010 Australia

**Keywords:** COVID-19, Excess mortality, Home deaths, Hospital deaths, United States, Place of death

## Abstract

Multiple cause of death data allow for a more granular analysis of the pandemic’s impact on mortality from non-COVID-19 causes of death compared with analysis of a single underlying cause of each death because they are often a co-morbidity of COVID-19. This study estimates excess mortality in the US of non-COVID-19 causes measured as a multiple cause (reported anywhere on the death certificate). Death registration data were used to conduct Poisson regressions of 24 non-COVID-19 causes to estimate expected age-standardized death rates and excess mortality in 2020–21, including by place of death. The ratio of COVID-19 mortality (as underlying or contributing cause) to excess mortality of each non-COVID-19 cause was calculated. During 2020–21, 21 of 24 non-COVID-19 causes exhibited excess mortality, highest for pneumonia (males 126.5%; 95% confidence interval 116.6–136.9%), other respiratory diseases (males 45.2%; 42.2–48.2%), other kidney diseases (males 45.0%, 37.8–52.0%), diabetes (females 38.3%, 32.8–43.4%) and hypertensive heart disease (females 28.9%, 22.8–33.9%). Suicide and influenza mortality was lower than expected. The ratio of COVID-19 (underlying cause) to excess mortality was 74.2% (69.2–79.7%) for men and 68.7% (63.5–75.0%) for women, was significantly higher for hospital (over 90%) than home (less than 16%) deaths and varied greatly between causes. The pandemic led to excess mortality for almost all non-COVID-19 causes in the US when measured as a multiple cause, being particularly high in several non-communicable diseases that increase the risk of dying from COVID-19. COVID-19 appears underreported for home deaths due to its low ratio to excess mortality in this setting.

**Supplementary Information:**

The online version contains supplementary material available at 10.1007/s10654-025-01214-z.

## Introduction

The COVID-19 pandemic has had a profound impact on mortality in the United States. In 2020–21, there were approximately one million excess deaths, or 17% more, compared to what was expected based on historical trends, leading to a fall in life expectancy unseen for several decades [1–3]. Deaths due to causes not reported as COVID-19 (non-COVID-19 causes) were also significantly impacted by the pandemic, comprising approximately 20% of excess mortality in 2020–21 when measured as the underlying cause of death (UC), or the cause initiating the chain of events leading to death [1].

There are several reasons why mortality from non-COVID-19 causes may have been impacted by the pandemic, either as the UC or as a contributing cause (CC), i.e. reported on the death certificate but not the UC. Firstly, COVID-19 commonly has multiple co-morbidities, which can lead to an increase in mortality from non-COVID-19 causes, including due to under-reporting of COVID-19 [4]. The long-term effects of COVID-19 can also increase people’s risk of dying from other diseases [5–7]. However, there is a lack of understanding of the extent to which under-reported COVID-19 deaths may have influenced trends in non-COVID-19 mortality during the pandemic [8]. Secondly, measures implemented to contain COVID-19, such as lockdowns, may have reduced the spread of other infectious diseases [9]. Thirdly, a strained health system during the pandemic may have negatively impacted on the ability of people to receive health care and increased their risk of dying from non-COVID-19 causes [10]. Finally, non-COVID-19 causes of death may have fallen because people who would otherwise have died from that cause instead died from COVID-19. Analysis of excess mortality from non-COVID-19 causes of death, including where they were a co-morbidity of COVID-19, can help understanding of these mortality dynamics during the pandemic.

Existing evidence shows that the COVID-19 pandemic had a varied impact on mortality from different diseases in the US [11, 12]. Several diseases were reported to have had excess deaths in 2020 and 2021, such as pneumonia, diabetes and kidney disease, while there were a number of diseases in which deaths were lower than expected, such as influenza and chronic lower respiratory disease [13–16]. However, such studies have only focused on a single UC; a more granular understanding of non-COVID-19 mortality during the pandemic can be gained by analyzing such causes whether they were a co-morbidity of COVID-19 or not.

Analysis of non-COVID-19 causes of death according to whether they were a co-morbidity of COVID-19 can be conducted by using multiple cause of death data, which comprise all causes reported on a death certificate, rather than relying on a single UC, as is commonly used in official statistics. Multiple cause of death data have become important during the pandemic because more causes have been reported per death than in previous years due to COVID-19’s interaction with other causes in leading to death [17]. These data allow calculation of, for non-COVID-19 causes of death, the ratio of (1) mortality where COVID-19, as either the UC or a CC, was reported with the non-COVID-19 cause, to (2) excess mortality from that non-COVID-19 cause. This ratio can help identify the extent to which changes in mortality from a non-COVID-19 cause occurred as a co-morbidity of COVID-19 or not, which is valuable in understanding whether cause-specific mortality changed during the pandemic due to the various factors described above. Multiple cause of death data can also help overcome the likely distortion of non-COVID-19 UC mortality trends due to the World Health Organization’s specific guidelines that recommended COVID-19 be the UC to improve the monitoring of mortality trends related to the pandemic; the impact of such guidelines would be to reduce non-COVID-19 UC but not necessarily as a CC.[17] Analysis of multiple cause of death data by their place of occurrence is also important to understand the extent to which COVID-19 was underreported for deaths that occur outside of healthcare facilities; that is, we would expect the proportion of cause-specific excess mortality where COVID-19 was listed as a multiple cause of death, including both the UC and a CC, to be lower for home deaths than for hospital deaths [18].

In this study, we measure excess mortality from 24 non-COVID-19 causes of death in the US during the pandemic (2020–21) using multiple cause of death data. For each non-COVID-19 cause, we also measure the ratio of COVID-19 mortality (either reported with the non-COVID-19 cause as the UC or CC) to excess mortality, to assess the contribution of COVID-19 to changes in mortality in the pandemic. We also analyze cause-specific excess mortality by place of death to understand underreporting of COVID-19 mortality. Although the worst of this pandemic is over, the outcomes of this study can provide important insights into understanding the role of direct and indirect factors on excess mortality during the COVID-19 pandemic and assist in formulating policies to reduce mortality in future pandemics.

## Methods

This study uses the US Centers for Disease Control and Prevention (CDC) Mortality Multiple Cause Files data from 2010 to 2021. The data comprise all registered deaths in the US, and includes information on all diseases and conditions that are reported on the medical certificate of cause of death (or death certificate) [19]. The certificate mandates the certifier (mainly a physician, but can also be a coroner, a nurse practitioner or another designated agent) to report the sequence of diseases or conditions that resulted in death, which is recorded in Part 1 of the certificate. Part 2 captures other significant conditions that contributed to the cause of death [4]. A multiple cause (MC) is defined where the cause is reported anywhere in either Part 1 or Part 2 of the certificate. Other variables in the dataset that were used in the analysis included the sex of death, age of death, date of death, and place of death (focusing on home and hospital deaths). The dataset did not include detailed geographic data such as county or state of residence, so it was not possible to identify any spatial differences in the analysis.

Twenty-four causes of death other than COVID-19 were classified using the International Classification of Diseases 10th version (ICD-10) [20]. These are either leading causes of death or those which are demonstrated or expected to be affected by the pandemic—see supplementary Text 1 for a listing of these causes and their ICD-10 codes, as well as the ICD-10 codes used for COVID-19. Each non-COVID-19 cause of death was analyzed as a MC, being counted as one death irrespective of whether it was the UC or a CC (reported on the death certificate in Part 1 or Part 2 but not the UC). Hence, a single death could be reported as a MC for more than one non-COVID-19 cause; for example, if diabetes and chronic kidney disease were both reported on the same death certificate, one diabetes MC death and one chronic kidney disease death would be counted. This “double counting” of deaths across different causes is not relevant for this study given that we are analyzing mortality from each non-COVID-19 cause of death separately as MC (for the reasons explained earlier), rather than aggregating them to equal all-cause mortality which can be done with a single UC for each death.

Poisson regression was used to predict expected mortality for each of the 24 non-COVID-19 causes (measured as a MC) for 2020, 2021, and both years combined. The regressions were conducted using 2010–19 historical data, being run separately for each combination of non-COVID-19 cause and sex, and, for the additional analyses, place of death. A different reference period of 2016–19 was also used to assess sensitivity of the results, however results from models using the 2010–19 reference period showed greater stability in results across the range of causes of death due to being based on data from a broader time period. The Poisson regression model used covariates of year of death, a third-degree polynomial of month of death, and age group, offset by the natural log of population. Population data were obtained from the US Census Bureau [21]. The model was specified as below:

$$\text{ln}\left({D}_{amy}\right)=\text{ln}\left({P}_{amy}\right)+ {\beta }_{0}+ {\beta }_{1}{Year}_{y}+ {\beta }_{2}{Month}_{m}+ {\beta }_{3}{Month}_{m}^{2}+ {\beta }_{4}{Month}_{m}^{3}+ {\beta }_{5-22}{Age}_{a}$$

where *D* is deaths, *P* is population, *a* is five-year age group (0–4 to 85+ years), *m* is month and *y* is year. To model the annual trend, we assessed both linear and cubic specifications of the *year* variable. The linear model demonstrated narrower uncertainty intervals, indicating a more robust fit, and was therefore selected as the preferred approach for this analysis.

The number of expected deaths (by 5-year age group) for 2020, 2021 and both years combined were estimated from each regression and were then used to calculate expected age-standardized mortality rates (per 100,000) for each of the 24 non-COVID-19 causes of death, using the 2010 US population as the standard population. Actual age-standardized mortality rates for each of the 24 non-COVID-19 were also calculated for 2020, 2021 and both years using observed data. Excess mortality was measured as both absolute and relative values. Absolute excess mortality was calculated as the difference between the actual age-standardized death rate and the expected age-standardized mortality rate [22]. Relative excess mortality was calculated by dividing the actual age-standardized mortality rate by the expected age-standardized death rate and subtracting 1, and measured as a percentage [22]. 95% uncertainty intervals of excess mortality were calculated using 1000 simulations that incorporated uncertainty in the expected age-standardized death rate from the regression model and stochastic uncertainty in the actual age-standardized death rate.

The contribution of COVID-19 to excess mortality was calculated as the ratio of the actual age-standardized death rate for each non-COVID-19 cause of death MC with COVID-19, either reported as the UC or as a CC, divided by the age-standardized excess mortality rate for that non-COVID-19 cause. Excess mortality not accounted for by COVID-19 UC or CC was classified as “No COVID”. In certain cases, the combined COVID-19 mortality rate for UC and CC was higher than the excess mortality rate, resulting in a lower-than-expected number of deaths for that non-COVID-19 cause excluding COVID-19 as a co-morbidity. Further analyses were performed independently based on the place of death (using the same methods as described above) to evaluate the underreporting of COVID-19 in home deaths. All analysis were conducted with Stata 17.0 [23].

Data were obtained from a publicly available database, so ethics approval was not required.

## Results

**Fig. 1:**
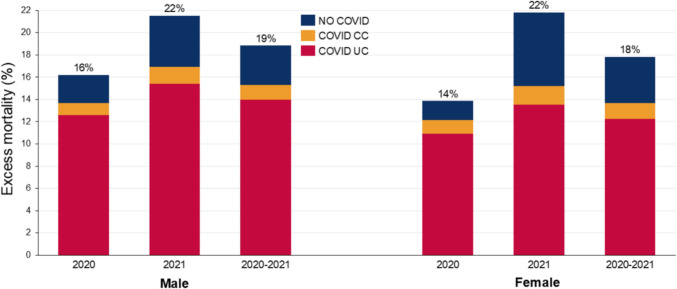
Excess mortality and ratio of COVID-19 (UC and CC) to excess mortality, all causes, by sex, US, 2020, 2021 and 2020–21. Uncertainty intervals shown in supplementary Tables 1-6. Percentage figure refers to overall excess mortality for all causes: the sum of COVID UC, COVID CC and No COVID.

During 2020–2021, all-cause excess mortality among men in the US was 18.8% (95% confidence interval (CI): 17.3, 20.5%) higher than expected, while among women it was 17.8% (16.1, 19.6%) (Fig. 1, supplementary Tables 1 and 2). The ratio of COVID-19 UC to excess mortality was 74.2% (69.2, 79.7%) for men and 68.7% (63.5, 75.0%) for women and as a CC was 6.9% (6.4, 7.4%) for men and 8.1% (7.5, 8.8%) for women. Excess mortality for males in 2020 was 16.2% (14, 18.4%), which increased to 21.5% (19.3, 23.7%) in 2021 (Fig. 1, supplementary Tables 3 and 5). The ratio of COVID-19 UC to the excess mortality rate was lower in 2021 (71.5% (66.1, 78.4%)) than 2020 (77.8% (69.7, 88.4%)), although with overlapping 95% CIs, while the COVID-19 CC ratio was steady. Excess mortality for females in 2020 was slightly lower at 13.9% (11.6, 16.3%) and also rose to be similar at 21.8% (19.3, 24.1%) in 2021 (Fig. 1, supplementary Tables 4 and 6). The ratio of COVID-19 UC to the excess mortality rate for females fell from 78.9% (68.6, 92.3%) 2020 to 62.1% (57.3, 68.8%) in 2021, and the ratio for COVID-19 CC also fell over these years. The observed and expected number of deaths for each cause, and number of COVID-19 UC, COVID-19 CC, and excess deaths for 2020 and 2021, is in supplementary Table 7.

**Fig. 2:**
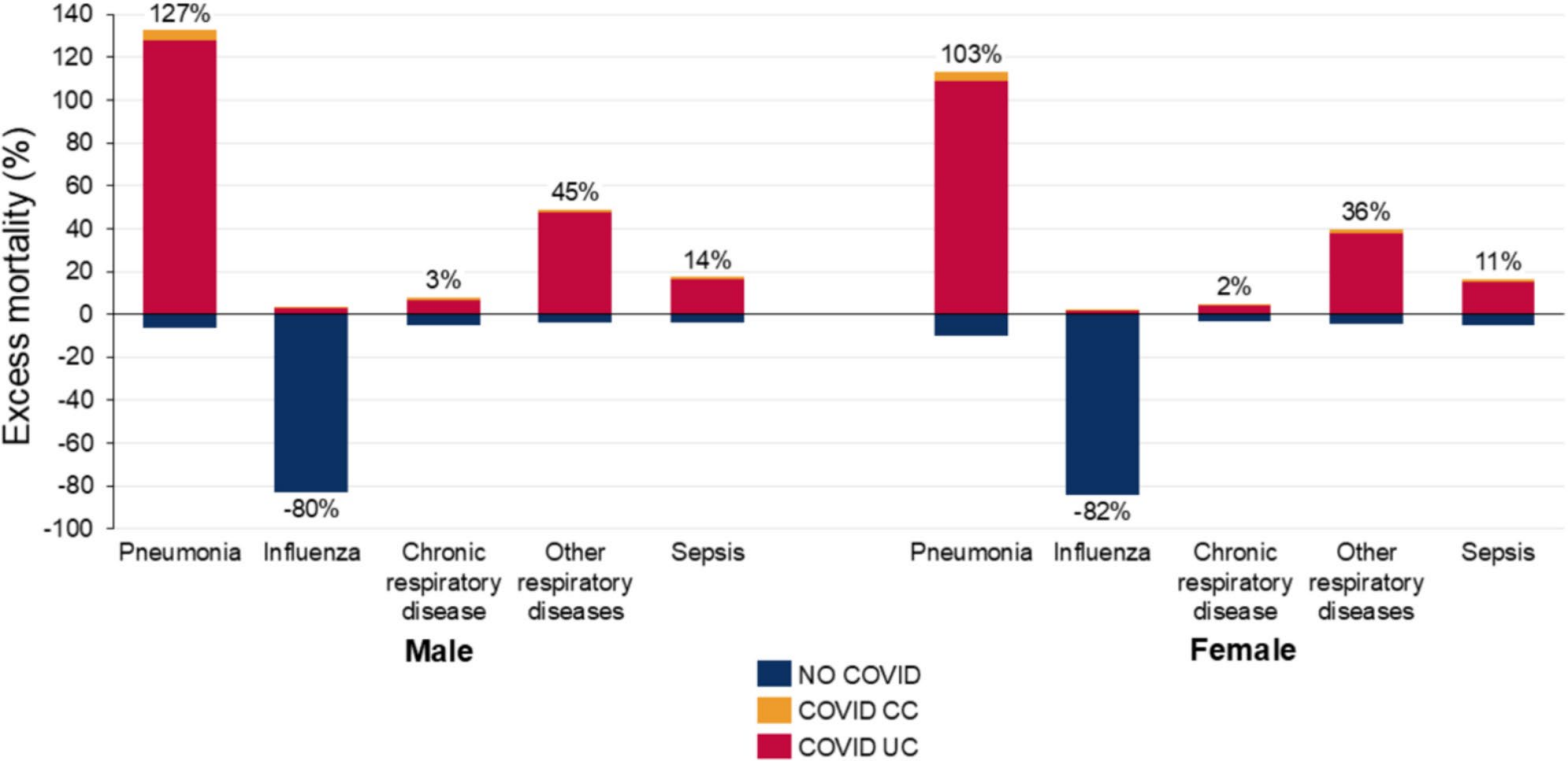
Excess mortality and ratio of COVID-19 (UC and CC) to excess mortality, infectious and respiratory diseases, by sex and cause of death, US, 2020–2021. Uncertainty intervals shown in supplementary Tables 1 and 2. Percentage figure refers to overall excess mortality for that cause: the sum of COVID UC, COVID CC and No COVID.

Of the 24 non-COVID-19 causes of death, for 21 causes the mortality rate in 2020–21 was higher than expected (based on MC) (supplementary Tables 1 and 2). Only influenza, suicide and lung cancer mortality was lower than expected (supplementary Figs. 1–4). During 2020–21, the highest excess mortality rate of the 24 causes was for pneumonia at 126.5% (116.6, 136.9%) for men and 103.3% (92.3, 114.3%) for women, respectively (Fig. 2, supplementary Tables 1 and 2). There was higher excess mortality from pneumonia in 2021 (149.3% (134.2, 166.7%) for males, 125.3% (109.3, 143.1%) for females) than 2020 (104.9% (91.7, 117.6%) for males, 81.2% (68, 93.7%) for females) (supplementary Fig. 1, supplementary Tables 3–6). During 2020–21, the ratio of COVID-19 UC to excess mortality for pneumonia exceeded 100% for both males and females, indicating that the age-standardized death rate of pneumonia with COVID-19 as the UC was greater than the total excess age-standardized death rate for pneumonia; this can occur because the pneumonia age-standardized death rate without COVID-19 (either as UC or CC) was lower than expected. Across the same period, COVID-19 CC accounted for less than 5% of the excess mortality in both sexes.

In stark contrast, the mortality rate for influenza in 2020–2021 was 79.8% lower than expected (− 86.2, − 73.8%) for men and 82.2% lower (− 88.0, − 76.5%) for women, even lower in 2021 than 2020 (Fig. 2; supplementary Fig. 1). The excess mortality rates for other respiratory diseases in 2020–21 were 45.2% (42.2, 48.2%) for men and 35.6% (32.4, 39%) for women (Fig. 2), respectively, being higher in 2021 than 2020 (supplementary Fig. 1). Excess mortality from chronic respiratory diseases was much lower, being less than 5% for each sex in 2020–21. It was over 10% for sepsis in 2020–21, being much higher in 2021 than 2020. The ratio of COVID-19 UC to excess mortality for chronic respiratory diseases exceeded 200% and for other respiratory diseases, and sepsis was greater than 100% during 2020–21.

**Fig. 3:**
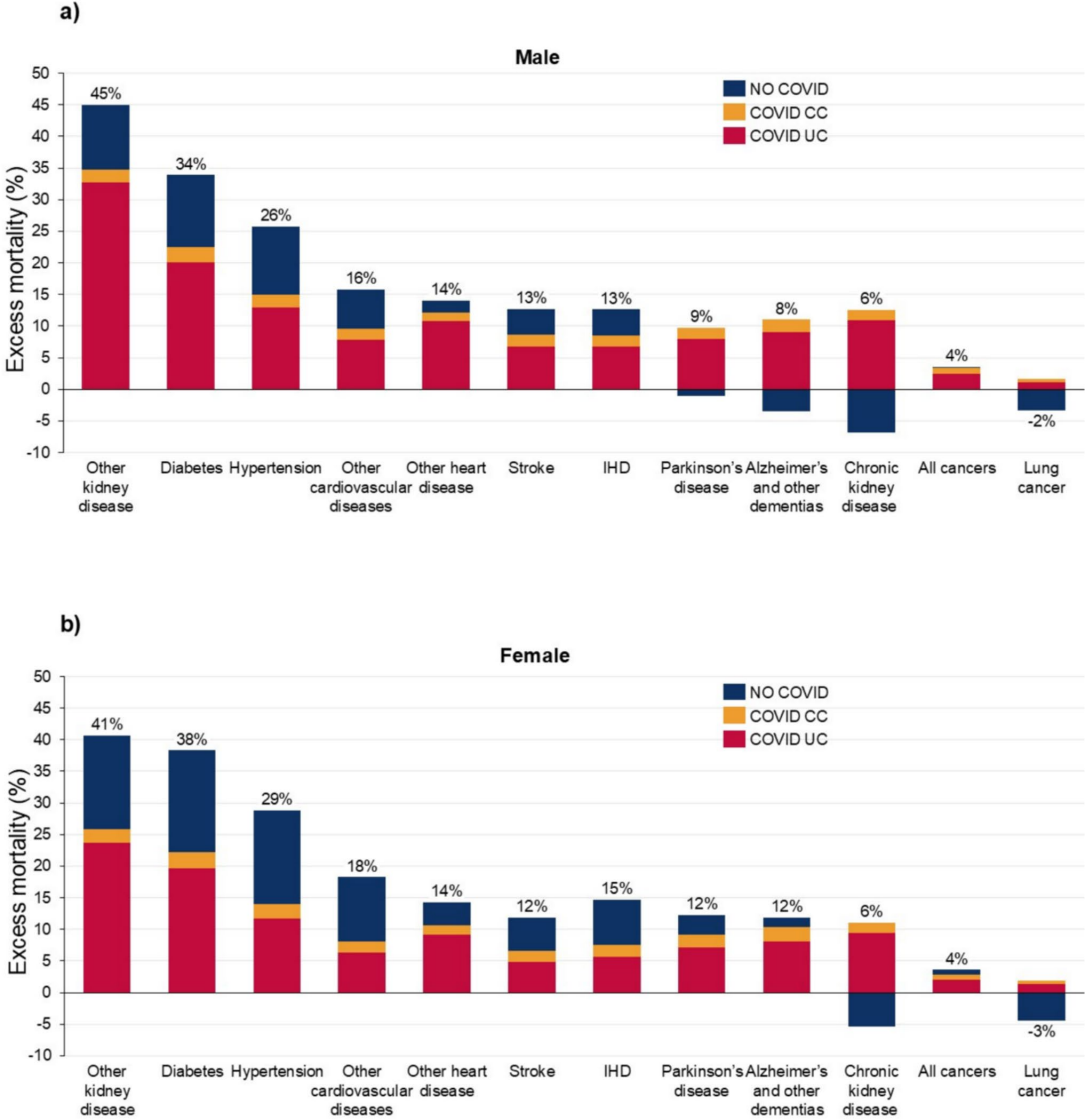
Excess mortality and ratio of COVID-19 (UC and CC) to excess mortality, non-communicable diseases, by sex and cause of death, US, 2020–2021. Uncertainty intervals shown in supplementary Tables 1 and 2. Percentage figure refers to overall excess mortality for that cause: the sum of COVID UC, COVID CC and No COVID.

Among non-communicable diseases, the highest excess mortality in 2020–21 was observed in other kidney disease (males 45% (37.8, 52%); females 40.7% (33.2, 47.9%)), followed by diabetes (males 33.9% (29.3, 38.5%); females 38.3% (32.8, 43.4%)), hypertensive heart disease (males 25.7% (22, 29.6%); females 28.9% (22.8, 33.9%)), and other cardiovascular diseases (males 15.8% (11.1, 21.1%); females 18.3% (11.7, 24.5%)) (Fig. 3, supplementary Tables 1 and 2). The three non-communicable diseases with the lowest excess mortality among males were chronic kidney disease (males 5.7% (0.5, 10.7%); females 5.7% (0.5, 11.6%)), all cancers (males 3.5% (1.7, 5.5%); females 3.6% (1.7, 5.5%)), and lung cancer (males − 1.7% (− 5.4, 2.0%); females − 2.5% (− 6.1, 1.2%)). Other heart diseases, stroke, IHD, Parkinson’s and Alzheimer's and other dementias had similar levels of excess mortality for males and females, being between 8% and 15%.

Also for non-communicable diseases in 2020–21, the ratio of COVID-19 UC to excess mortality for Alzheimer's and other dementias and chronic kidney disease was over 100% in males but only for chronic kidney disease in females. The ratio of COVID-19 UC to excess mortality was over 50% for other kidney disease, diabetes, hypertensive heart disease (males only), other heart disease, stroke (males only), IHD (males only) and all cancers (Fig. 3, supplementary Tables 1 and 2). For all non-communicable diseases, except Parkinson’s disease, Alzheimer’s disease and chronic kidney disease (males only), there was higher excess mortality in 2021 than 2020 (supplementary Figs. 2–3). The ratio of COVID-19 UC to excess mortality rates did not show significant changes between 2020 and 2021, except for a decline for other kidney diseases for males, a notable increase for male Parkinson’s disease, and a significant decline for both male and female all cancers. In 2020–21, the ratios of COVID-19 CC to excess mortality for Parkinson’s disease (males only), Alzheimer's and other dementias (males only), chronic kidney disease, all cancers and lung cancer exceeded 20%, and were lower for other causes.

**Fig. 4:**
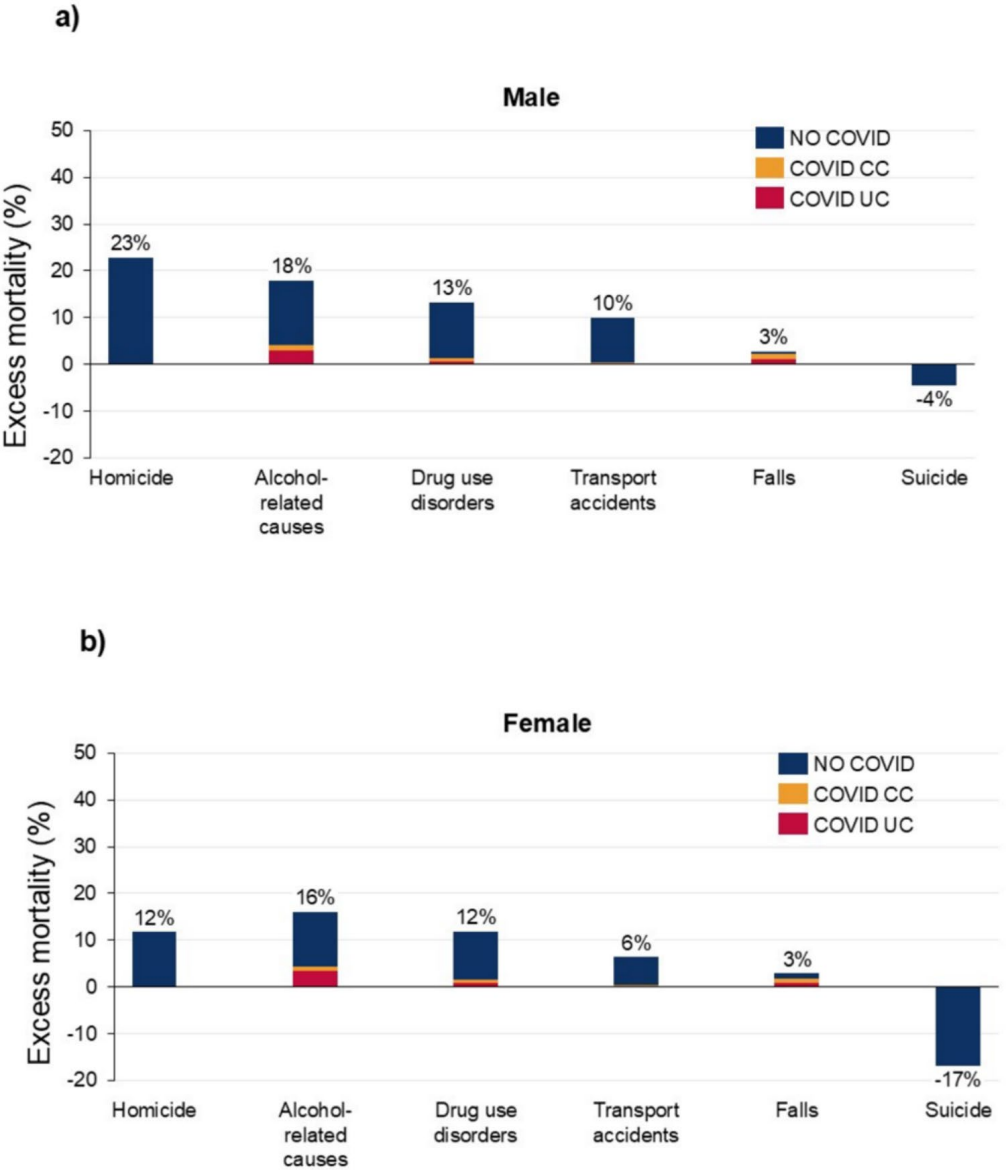
Excess mortality and ratio of COVID-19 (UC and CC) to excess mortality, other causes, by sex and cause of death, US, 2020–2021. Uncertainty intervals shown in supplementary Tables 1 and 2. Percentage figure refers to overall excess mortality for that cause: the sum of COVID UC, COVID CC and No COVID.

Among the six other causes in 2020–21, the highest excess mortality was observed for homicide (males 22.8% (17.0, 28.7%); females 11.7% (6.6, 16.7%)). This was followed by alcohol-related conditions (males 18.0% (15.3, 20.6%); females 16.1% (12.8, 19.3%)), drug use disorders (males 13.2% (8.9, 17.9%); females 11.9% (7.6, 16.1%)), and transport accidents (males 10.0% (7.7, 12.0%); females 6.3% (3.8, 9.1%)). For falls, both males and females had an excess mortality rate of 2.8% (males (− 3.3, 9.9%); females (− 5.4, 11.4%)). Suicide had less deaths than expected (males − 4.4% (− 5.9, − 2.6%); females − 16.7% (− 19.3, − 14.1%)) (Fig. 4, supplementary Tables 1 and 2). The ratio of COVID-19 UC to excess mortality was over 30% for falls among males and exceeded 20% for females. For alcohol-related causes, the ratio exceeded 15% for both males and females. For homicide, drug use disorders, transport accidents, and suicide, the ratios were all below 7%.

**Fig. 5:**
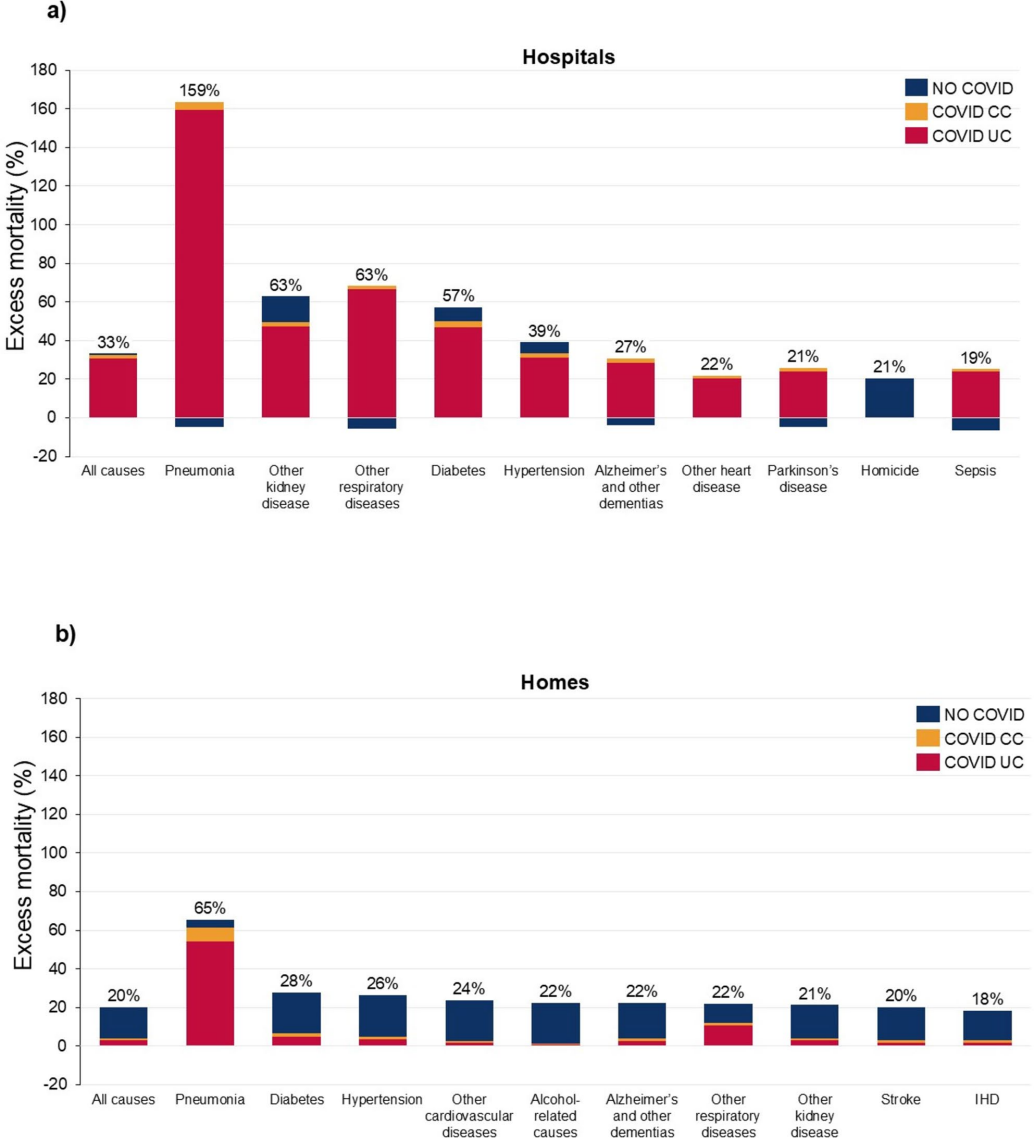
Top 10 causes of death according to highest excess mortality, hospital deaths and home deaths, males, US, 2020–2021. Uncertainty intervals shown in supplementary Tables 8 and 10. Percentage figure refers to overall excess mortality for that cause: the sum of COVID UC, COVID CC and No COVID.

**Fig. 6:**
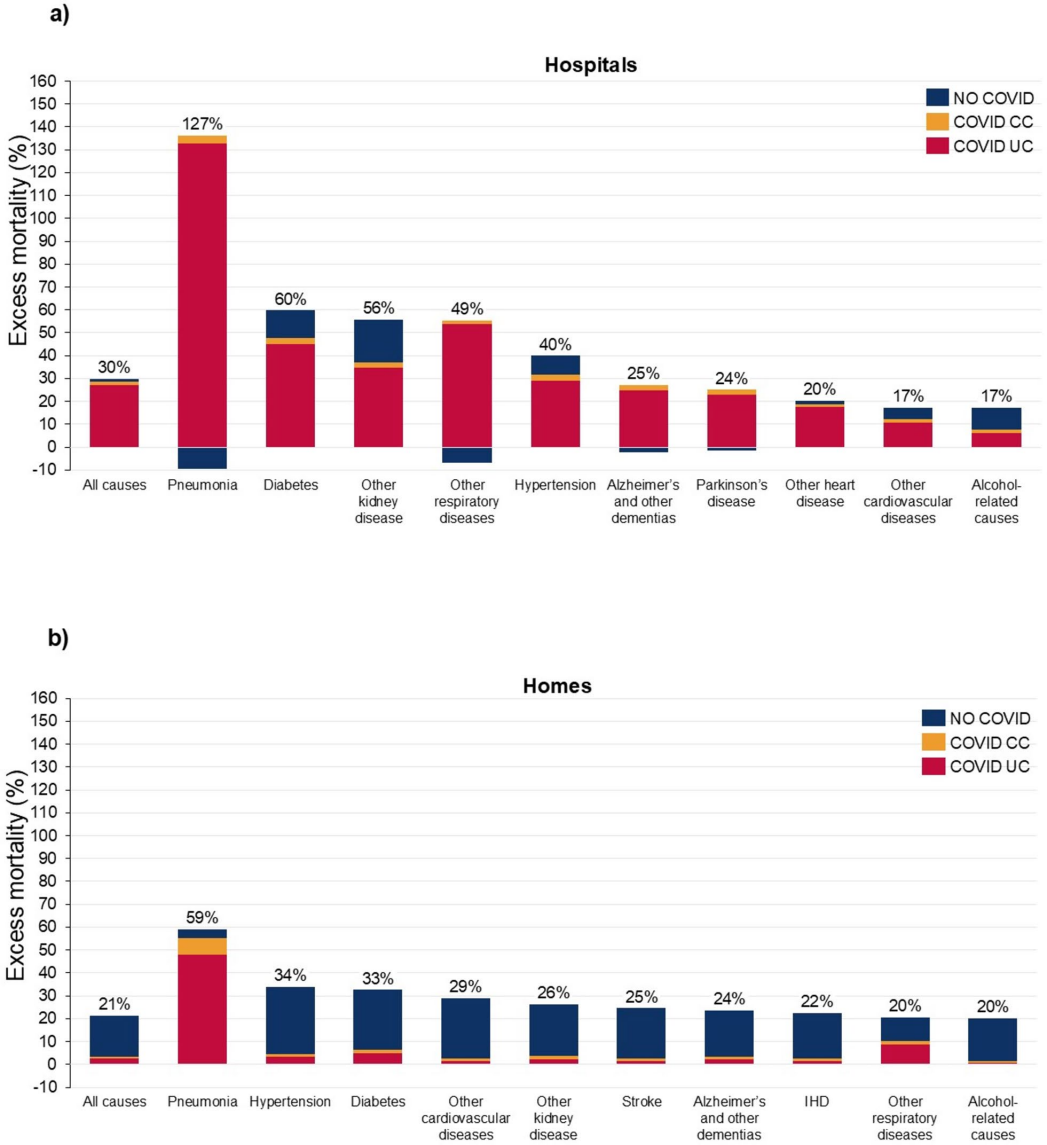
Top 10 causes of death according to highest excess mortality, hospital deaths and home deaths, females, US, 2020–2021. Uncertainty intervals shown in supplementary Tables 9 and 11. Percentage figure refers to overall excess mortality for that cause: the sum of COVID UC, COVID CC and No COVID.

Table 1 shows that, following an increase in home deaths relative to hospitals deaths in the pre-pandemic period, there was a sharp increase in hospital deaths and decline in hospice/nursing home deaths in 2020–21. All-cause mortality rates in 2020–21 were higher for hospital (males 33.2% (31.4, 35.1%), females 29.8% (27.9, 31.7%)) than home deaths (males 20.2% (18.4, 22.1%), females 21.4% (19.1, 23.6%)), and COVID-19 UC made a much greater contribution to excess mortality rates for hospital deaths (males 92.9% (89.1, 96.9%), females 90.7% (86.6, 95.5%)) than for home deaths (males 15.6% (14.5, 16.9%), females 12.5% (11.6, 13.8%)) (Figs. 5, 6). Excess mortality from pneumonia, other kidney diseases, other respiratory diseases and diabetes was far higher in hospitals than in homes, being more than double for some causes. For most causes of death, the ratio of COVID-19 UC to excess mortality in hospitals was over 90%, whereas for all causes for home deaths, except for pneumonia and other respiratory diseases, it was less than 20% of excess home deaths. Regardless of whether deaths occurred in hospitals or at home, COVID-19 CC was less than 10% of excess mortality for most causes, with the exception of pneumonia.

**Table 1:** Place of death (%), US, 2010–21

Place of death	2010	2011	2012	2013	2014	2015	2016	2017	2018	2019	2020	2021
Home	27.2	27.5	28.3	28.9	29.4	29.7	30.4	30.7	31.3	31.6	33.3	33.5
Hospital	40.0	39.1	38.0	37.3	36.8	36.3	35.9	35.5	35.1	34.8	36.3	39.2
Hospice/ nursing home	25.6	26.2	26.4	26.2	27.0	27.5	27.0	27.1	26.8	26.6	23.4	20.0
Other	7.2	7.2	7.3	7.6	6.7	6.5	6.7	6.7	6.8	7.0	7.1	7.3

## Discussion

Non-COVID-19 mortality in the US experienced a significant rise during the first two years of the COVID-19 pandemic, 2020 and 2021. Mortality rates for nearly all causes of death increased during this period, with notable exceptions being influenza, suicide, and lung cancer, which saw declines. A significant portion of the rise in non-COVID-19 deaths in the U.S. in 2020 and 2021 was not directly attributed to reported COVID-19 mortality, and this proportion was much higher for home than hospital deaths.

Our study estimated 1.04 million excess all-cause deaths in 2020 and 2021, consistent with other studies whose estimates ranged from 0.97 to 1.13 million deaths [2, 24–27]. Among the 24 non-COVID-19 causes, pneumonia had the highest excess mortality and COVID-19 contributed to almost all of its excess mortality. However, our findings differ from previous analysis focused solely on the UC. For example, a US study reported a 2% lower-than-expected mortality for influenza and pneumonia between March and December 2020 when only the UC was considered, due COVID-19 commonly being the UC when pneumonia was the more proximate cause of death, and highlighting potential biases in analyses that only consider the UC [16]. Of other respiratory diseases, influenza deaths were far lower than expected, possibly due to misclassification as COVID-19 or pneumonia or, more likely, due to reduced influenza circulation during lockdowns [28]. Excess chronic respiratory disease mortality was only very small while being among the highest of all causes for other respiratory diseases and, for each, all excess mortality was contributed to by COVID-19. It is likely that having a respiratory disease increased people’s likelihood of developing severe COVID-19 and dying; the low excess mortality of chronic respiratory diseases suggests that some of this mortality may have been misclassified as COVID-19 or COVID-19 being correctly classified but chronic respiratory not reported on the death certificate [29].

Excess mortality was also high for some non-communicable diseases, exceeding 25% for other kidney disease, diabetes and hypertensive heart disease. These causes, and their common underlying risk factor of obesity, have been found to increase the risk of dying from COVID-19 [22, 30, 31]. For these and cardiovascular diseases, COVID-19 UC contributed to over half of excess mortality, demonstrating that analysis of these common causes of death as just the UC does not adequately demonstrate their trends in the pandemic. Mortality from cancers increased by a smaller amount than other causes and, for the short-term at least, belied concerns that reduced cancer screenings during the pandemic would adversely affect mortality significantly [32, 33].

For other causes, there was considerable excess mortality from homicide and transport accidents, confirming other research [34–36]. The rise in transport-related deaths can be attributed to riskier behaviors such as speeding and impaired driving on less congested roads, with reports suggesting a false sense of security on emptier streets [37, 38]. Alcohol-related and drug-related deaths also increased sharply, continuing their adverse trends in the previous decade [34, 39]. Furthermore, the economic recession, rising unemployment, and prolonged isolation during the pandemic led to increased psychological stress which, in some countries, was found to cause a rise in drug-related deaths [40, 41]. However, in contrast to other nations, the US saw a decline in suicide deaths, which previous research has found likely driven by increased social cohesion, expanded virtual mental health services, and government financial support, mitigating some of the pandemic's psychological and economic pressures [42–48].

Our analysis of excess mortality rates by place of death (hospital vs. home) revealed key insights into COVID-19's role in non-COVID-19 mortality. Most causes showed higher excess mortality in hospitals. This may be because during the pandemic, when hospital resources were stretched, severity of condition was a more important factor affecting whether people sought treatment in hospitals than in pre-pandemic times. Further analysis of hospice/nursing home deaths (data not shown), where 22% of deaths occurred, revealed that there was negative excess mortality of 6% for males and 12% for females, suggesting that many older and more frail residents were treated and ultimately died in hospitals to a greater extent than in previous years. However, these findings may not be generalizable, as healthcare access varied significantly across states in the US; many remote areas faced significant challenges in accessing healthcare resources during the pandemic. Those with better economic conditions were more likely to access medical assistance.[49] Unfortunately, the lack of state-level data limited further analysis, especially in relation to drawing conclusions about the impact on the health system and of different restrictions imposed by various jurisdictions.

The contribution of COVID-19 UC to excess mortality was significantly higher in hospitals than at home, while its impact as a CC was similar in both settings. This is attributable to several reasons. Firstly, many doctors, particularly pediatricians, tended to classify any hospital patient exhibiting COVID-19 symptoms, such as a fever, as having COVID-19 unless proven otherwise [50]. This would have led to an overestimation of COVID-19 infection cases recorded in hospitals, along with the guidelines for reporting COVID-19 as the UC described earlier. Secondly, hospital patients were more vulnerable to COVID-19 than those at home, as they typically had more severe conditions and also hospitals posed a higher transmission risk due to proximity and the concentration of COVID-19 cases [51]. Thirdly, for home deaths, the cause of death recorded in the death certificate relied heavily on the patient's medical history and family accounts. Confirming COVID-19 infection in the deceased required post-mortem examinations, which were only conducted for a small proportion of unusual deaths or when requested by family members [52]. As a result, the reporting of COVID-19 cases among individuals who died at home was likely underreported. Finally, excess non-COVID-19 home deaths may have resulted from restricted access to treatment during lockdowns.

This study has limitations other than those already mentioned. Firstly, this study is a quantitative data analysis using a large dataset focusing on a wide range of causes of death. A more focused study on specific causes of death (e.g., the precise role of COVID-19 in increasing the risk of mortality from a specific cause(s) beyond what can be concluded from what is reported on the death certificate) or the impacts of specific issues related to the pandemic (e.g. burdened health system, COVID-19 “displacing” mortality from specific non-COVID-19 causes) would provide additional insights. In addition to COVID-19 being underreported as a cause of death, the reporting for all causes may be affected by accuracy. This could result in misclassification of deaths between causes, for example cardiovascular diseases, but use of larger cause groups to overcome this issue would result in less detailed and informative results. Furthermore, the US’s mortality data have been found to be among the highest quality in the world [53]. Geographic analysis was not possible due to the lack of a state variable in the dataset, though previous studies showed the highest excess mortality in southern states during the pandemic’s first year [54]. Use of the the National Center for Health Statistics (NCHS) restricted mortality data files, which include state and county level data, would enhance the insights from such an analysis.

In conclusion, multiple cause of death data provides insights beyond what underlying cause data alone can reveal about non-COVID-19 causes of death, especially for assessing co-morbidities with COVID-19, as well as demonstrating the underreporting of COVID-19. We found that mortality trends in various non-COVID-19 causes were likely contributed by the higher mortality risk from COVID-19, lockdowns and the strained health system. Some of the practical implications of the findings for future pandemic could be to prioritise protection of high-risk groups, improve monitoring and reporting systems, prepare contingency plans and resource reserves in advance for non-COVID-19 diseases, and to emphasise the ensure continuity of healthcare to prevent disruption of health services. Future studies could consider and apply the methodology used in this study to comprehensively investigate the impact of COVID-19 on specific causes of death. Additionally, in-depth exploration of the factors contributing to underreporting and overreporting of COVID-19 should also be conducted, especially for specific diseases or disease groups, to assist in providing more evidence of the impact of the COVID-19 pandemic on mortality.

## Supplementary Information

Below is the link to the electronic supplementary material.

Supplementary file1 (DOCX 2228 KB)

## Data Availability

The multiple cause of death data file is available from the National Center for Health Statistics (https://www.cdc.gov/nchs/nvss/mortality_public_use_data.htm).

## Abbreviations

CC: Contributing cause (reported on the death certificate but not the underlying cause of death)
COVID-19: Coronavirus disease
COVID-19 CC: COVID-19 not the underlying cause of death but a contributing cause
COVID-19 UC: COVID-19 as the underlying cause of death
MC: Multiple cause of death
Non-COVID-19: Causes of death other than COVID-19
UC: Underlying cause of death
