# Supplementary material for: The impact of the pandemic on non-COVID-19 causes of death in the United States: a multiple cause of death analysis
Source: Eur J Epidemiol. 2025 Mar 19;40(4):463–74. doi: 10.1007/s10654-025-01214-z (PMC12145281; doi:10.1007/s10654-025-01214-z)
Supplement: Supplementary file 1 — Supplementary file1 (DOCX 2228 KB) [file 10654_2025_1214_MOESM1_ESM.docx]

**THE IMPACT OF THE PANDEMIC ON NON-COVID-19 CAUSES OF DEATH IN THE UNITED STATES: A MULTIPLE CAUSE OF DEATH ANALYSIS**

**SUPPLEMENTARY MATERIAL**

**Text 1** Causes of death and ICD-10 codes

Non-COVID-19 causes of death analyzed were:

1. Alzheimer's and other dementias (ICD-10 codes F00, F01, F03, G30, G31)

2. Ischemic heart disease (IHD) (I20-I25)

3. Stroke (I60-I69)

4. Other heart disease (I26-I51)

5. Hypertension (I10-I13)

6. Other cardiovascular diseases (I00-I09, I14-I19, I52-I59, I70-I99)

7. Diabetes (E10-E14)

8. Chronic kidney disease (N18)

9. Other kidney disease (N17, N19)

10. All cancers (C00-D44)

11. Lung cancer (C34)

12. Sepsis (A41)

13. Chronic respiratory disease (J40-J44)

14. Influenza (J09-J11)

15. Pneumonia (J12-J18)

16. Other respiratory diseases (J00-J06, J20-J39, J45-J98)

17. Parkinson's disease (G20)

18. Ill-defined causes (R00-R99)

19. Falls (W00-W19)

20. Homicide (X85-Y09)

21. Alcohol-related causes (F10, G31.2, K70, K74, K76.0-K76.2, K76.4-K76.9, X45, Y15)

22. Drug use disorders (F11-F16, F18-F19, X40-X44, Y10-Y14)

23. Transport accidents (V00-V99)

24. Suicide (X60-X84)

COVID-19 was identified and reported in the data using the ICD-10 codes U07.1 and U07.2.

**Fig. 1** Excess mortality and ratio of COVID-19 (UC and CC) to excess mortality, infectious and respiratory diseases, by sex and cause of death, US, 2020 and 2021

**
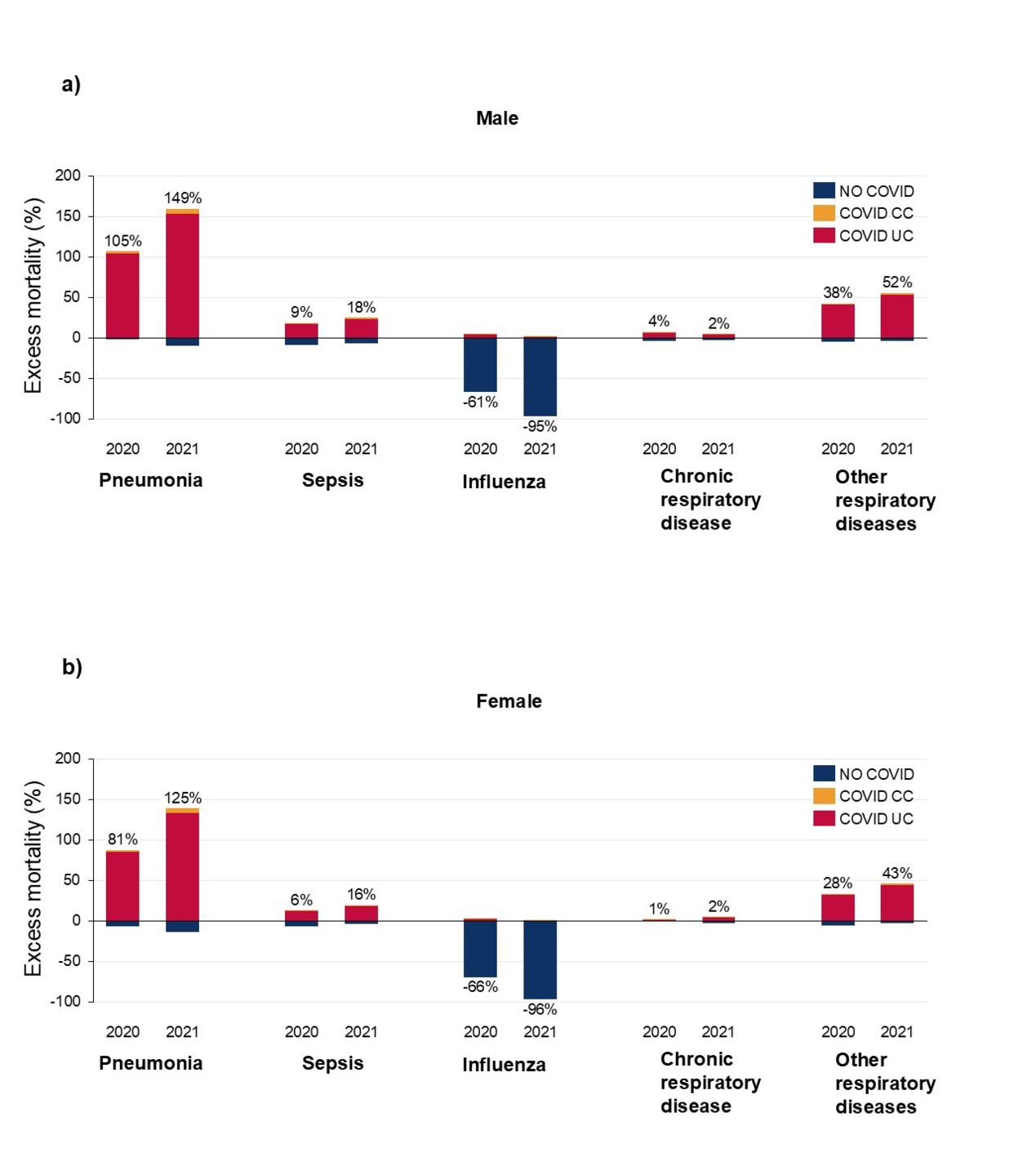
**

Uncertainty intervals shown in supplementary Tables 3-6

**Fig. 2** Excess mortality and ratio of COVID-19 (UC and CC) to excess mortality, non-communicable diseases, males, by cause of death, US, 2020 and 2021

**
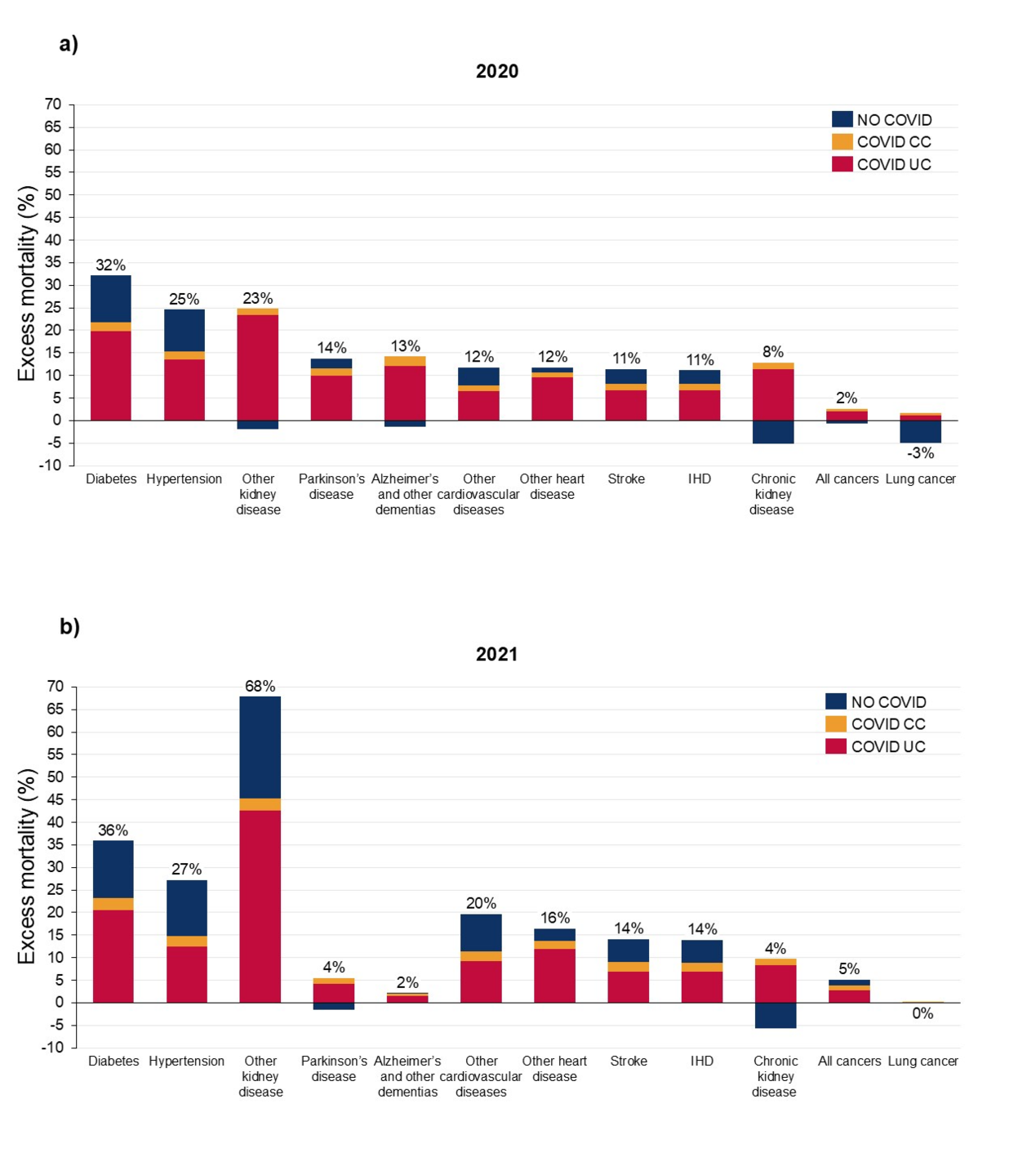
**

Uncertainty intervals shown in supplementary Tables 3 and 5

**Fig. 3** Excess mortality and ratio of COVID-19 (UC and CC) to excess mortality, non-communicable diseases, females, by cause of death, US, 2020 and 2021

**
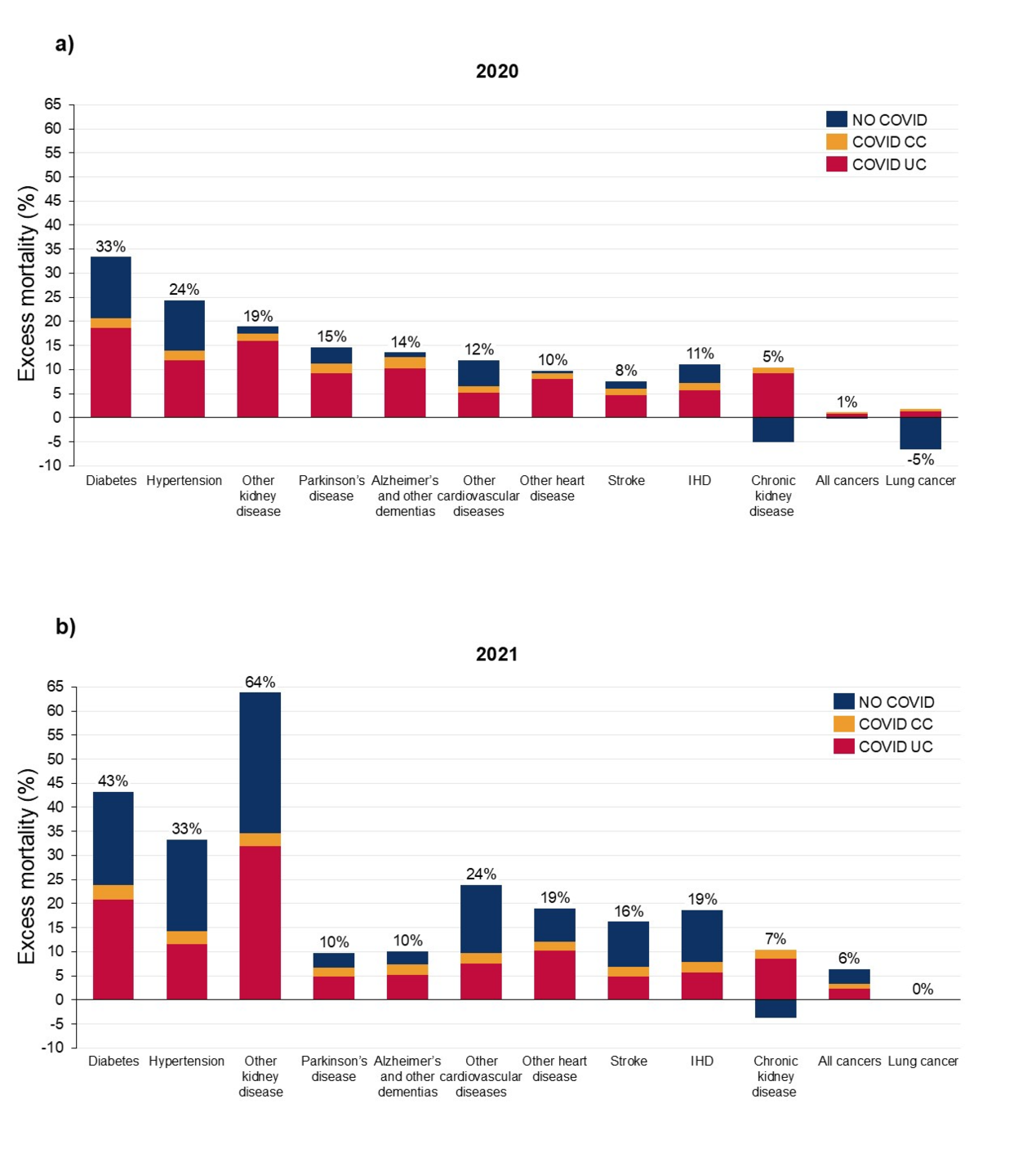
**

Uncertainty intervals shown in supplementary Tables 4 and 6

**Fig. 4** Excess mortality and ratio of COVID-19 (UC and CC) to excess mortality, other causes, by sex and cause of death, US, 2020 and 2021

**
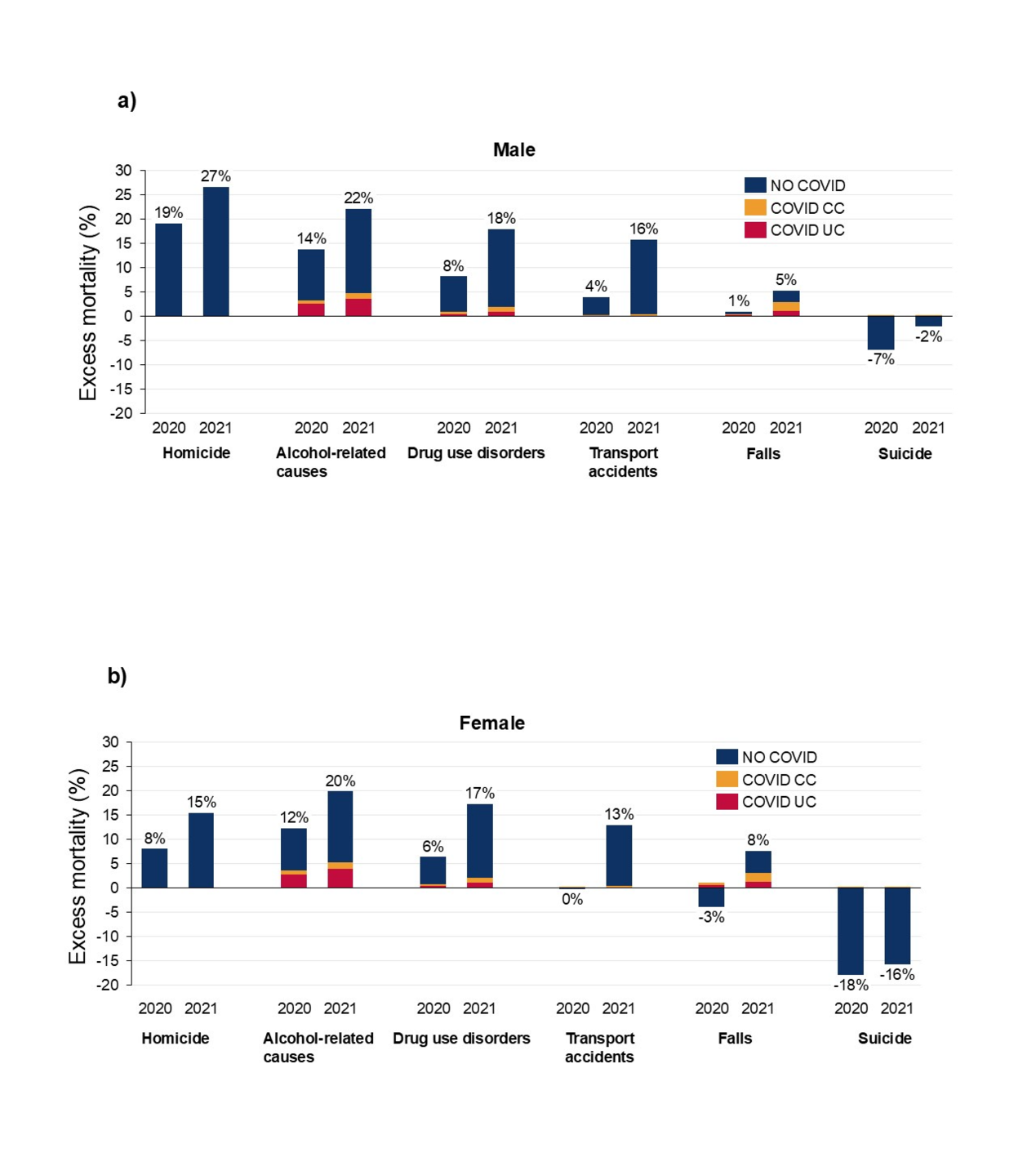
**

Uncertainty intervals shown in supplementary Tables 3-6

**Table 1** Excess mortality and ratio of COVID-19 (UC and CC) to excess mortality, males, US, 2020-2021

|  | **Excess mortality** | | | **Ratio of COVID-19 UC to excess mortality** | | | **Ratio of COVID-19 CC to excess mortality** | | | |
| --- | --- | --- | --- | --- | --- | --- | --- | --- | --- | --- |
| **Causes** | **%** | **95% CI** | | **%** | **95% CI** | | **%** | **95% CI** | | |
| **All causes** | 18.8 | 17.3 | 20.5 | 74.2 | 69.2 | 79.7 | 6.9 | 6.4 | 7.4 |  |
| **Pneumonia** | 126.5 | 116.6 | 136.9 | 101.3 | 97.7 | 105.1 | 3.5 | 3.4 | 3.7 |  |
| **Other respiratory diseases** | 45.2 | 42.2 | 48.2 | 104.9 | 100.3 | 110.2 | 3.8 | 3.6 | 4.0 |  |
| **Other kidney disease** | 45.0 | 37.8 | 52.0 | 72.8 | 66.0 | 82.4 | 4.5 | 4.1 | 5.1 |  |
| **Diabetes** | 33.9 | 29.3 | 38.5 | 59.4 | 54.2 | 66.4 | 6.8 | 6.2 | 7.6 |  |
| **Hypertensive heart disease** | 25.7 | 22.0 | 29.6 | 50.5 | 45.3 | 57.4 | 7.8 | 7.0 | 8.9 |  |
| **Homicide** | 22.8 | 17.0 | 28.7 | 0.1 | 0.1 | 0.1 | 0.4 | 0.3 | 0.5 |  |
| **Ill-defined** | 18.4 | 15.9 | 20.8 | 78.5 | 70.7 | 88.8 | 8.8 | 7.9 | 9.9 |  |
| **Alcohol-related causes** | 18.0 | 15.3 | 20.6 | 16.9 | 15.0 | 19.4 | 5.5 | 4.9 | 6.3 |  |
| **Other cardiovascular diseases** | 15.8 | 11.1 | 21.1 | 49.7 | 39.0 | 68.3 | 11.0 | 8.6 | 15.1 |  |
| **Other heart disease** | 14.0 | 11.8 | 16.3 | 76.4 | 67.2 | 89.0 | 10.3 | 9.1 | 12.0 |  |
| **Sepsis** | 13.8 | 9.3 | 18.0 | 148.0 | 117.5 | 210.9 | 8.6 | 6.9 | 12.3 |  |
| **Drug use disorders** | 13.2 | 8.9 | 17.9 | 5.5 | 4.2 | 7.9 | 5.3 | 4.0 | 7.6 |  |
| **Stroke** | 12.7 | 7.8 | 18.0 | 53.6 | 39.5 | 83.7 | 14.2 | 10.5 | 22.2 |  |
| **IHD** | 12.6 | 8.1 | 17.1 | 53.8 | 41.4 | 80.2 | 13.3 | 10.3 | 19.9 |  |
| **Transport accidents** | 10.0 | 7.7 | 12.0 | 1.5 | 1.3 | 1.9 | 2.2 | 1.9 | 2.8 |  |
| **Parkinson's disease** | 8.7 | 2.7 | 14.7 | 91.1 | 56.7 | 277.6 | 20.6 | 12.8 | 62.8 |  |
| **Alzheimer's and other dementias** | 7.6 | -2.1 | 17.6 | 118.8 | -444.8 | 918.9 | 26.8 | -100.4 | 207.3 |  |
| **Chronic kidney disease** | 5.7 | 0.5 | 10.7 | 192.2 | 99.2 | 1142.0 | 27.1 | 14.0 | 161.0 |  |
| **All cancers** | 3.5 | 1.7 | 5.5 | 70.8 | 46.2 | 149.0 | 23.8 | 15.5 | 50.0 |  |
| **Chronic respiratory disease** | 2.9 | -2.2 | 8.2 | 233.6 | -3874.9 | 2891.2 | 39.2 | -649.7 | 484.8 |  |
| **Falls** | 2.8 | -3.3 | 9.9 | 36.8 | -929.3 | 624.7 | 44.7 | -1130.3 | 759.8 |  |
| **Lung cancer** | -1.7 | -5.4 | 2.0 | -64.9 | -840.2 | 1006.7 | -27.9 | -361.2 | 432.8 |  |
| **Suicide** | -4.4 | -5.9 | -2.6 | -0.3 | -0.5 | -0.2 | -3.3 | -5.6 | -2.4 |  |
| **Influenza** | -79.8 | -86.2 | -73.8 | -3.4 | -4.8 | -2.2 | -0.6 | -0.9 | -0.4 |  |

95% CI: 95% confidence interval.

**Table 2** Excess mortality and ratio of COVID-19 (UC and CC) to excess mortality, females, US, 2020-2021

|  | **Excess mortality** | | | **Ratio of COVID-19 UC to excess mortality** | | | **Ratio of COVID-19 CC to excess mortality** | | |
| --- | --- | --- | --- | --- | --- | --- | --- | --- | --- |
| **Causes** | **%** | **95% CI** | | **%** | **95% CI** | | **%** | **95% CI** | |
| **All causes** | 17.8 | 16.1 | 19.6 | 68.7 | 63.5 | 75.0 | 8.1 | 7.5 | 8.8 |
| **Pneumonia** | 103.3 | 92.3 | 114.3 | 105.6 | 100.5 | 111.8 | 4.1 | 3.9 | 4.4 |
| **Other respiratory diseases** | 35.6 | 32.4 | 39.0 | 107.2 | 100.1 | 114.8 | 4.8 | 4.5 | 5.1 |
| **Other kidney disease** | 40.7 | 33.2 | 47.9 | 58.4 | 52.1 | 67.7 | 5.2 | 4.6 | 6.0 |
| **Diabetes** | 38.3 | 32.8 | 43.4 | 51.4 | 47.0 | 57.6 | 6.7 | 6.1 | 7.5 |
| **Hypertensive heart disease** | 28.9 | 22.8 | 33.9 | 40.7 | 36.0 | 48.9 | 7.9 | 7.0 | 9.5 |
| **Homicide** | 11.7 | 6.6 | 16.7 | 0.2 | 0.1 | 0.3 | 0.8 | 0.6 | 1.3 |
| **Ill-defined** | 21.2 | 18.0 | 24.3 | 55.9 | 49.8 | 64.1 | 8.7 | 7.8 | 10.0 |
| **Alcohol-related causes** | 16.1 | 12.8 | 19.3 | 20.5 | 17.5 | 25.1 | 6.8 | 5.8 | 8.3 |
| **Other cardiovascular diseases** | 18.3 | 11.7 | 24.5 | 34.8 | 27.4 | 51.5 | 9.4 | 7.4 | 14.0 |
| **Other heart disease** | 14.3 | 11.4 | 17.2 | 63.5 | 54.2 | 78.0 | 10.5 | 9.0 | 12.9 |
| **Sepsis** | 11.2 | 7.2 | 15.4 | 135.1 | 102.1 | 204.6 | 10.7 | 8.1 | 16.2 |
| **Drug use disorders** | 11.9 | 7.6 | 16.1 | 6.5 | 5.0 | 9.8 | 5.6 | 4.3 | 8.5 |
| **Stroke** | 11.8 | 5.7 | 18.5 | 40.9 | 27.8 | 80.6 | 14.5 | 9.9 | 28.6 |
| **IHD** | 14.7 | 9.0 | 20.7 | 38.8 | 28.9 | 60.3 | 12.5 | 9.3 | 19.4 |
| **Transport accidents** | 6.3 | 3.8 | 9.1 | 1.8 | 1.3 | 3.0 | 3.5 | 2.5 | 5.8 |
| **Parkinson's disease** | 12.2 | 4.8 | 19.5 | 58.1 | 38.7 | 139.2 | 16.3 | 10.9 | 39.1 |
| **Alzheimer's and other dementias** | 11.8 | 1.3 | 21.9 | 68.2 | 38.7 | 436.0 | 19.9 | 11.3 | 127.3 |
| **Chronic kidney disease** | 5.7 | 0.5 | 11.6 | 164.7 | 81.8 | 909.0 | 28.5 | 14.2 | 157.6 |
| **All cancers** | 3.6 | 1.7 | 5.5 | 55.1 | 36.8 | 115.3 | 22.3 | 14.9 | 46.7 |
| **Chronic respiratory disease** | 1.7 | -3.2 | 6.8 | 247.0 | -3594.0 | 4753.7 | 46.3 | -673.1 | 890.3 |
| **Falls** | 2.8 | -5.4 | 11.4 | 26.9 | -455.3 | 556.8 | 34.9 | -591.2 | 723.0 |
| **Lung cancer** | -2.5 | -6.1 | 1.2 | -52.8 | -582.7 | 617.5 | -24.3 | -268.3 | 284.4 |
| **Suicide** | -16.7 | -19.3 | -14.1 | -0.1 | -0.1 | -0.1 | -0.9 | -1.1 | -0.8 |
| **Influenza** | -82.2 | -88.0 | -76.5 | -1.9 | -2.7 | -1.2 | -0.4 | -0.5 | -0.2 |

95% CI: 95% confidence interval.

**Table 3** Excess mortality and ratio of COVID-19 (UC and CC) to excess mortality, males, US, 2020

|  | **Excess mortality** | | | **Ratio of COVID-19 UC to excess mortality** | | | **Ratio of COVID-19 CC to excess mortality** | | |
| --- | --- | --- | --- | --- | --- | --- | --- | --- | --- |
| **Causes** | **%** | **95% CI** | | **%** | **95% CI** | | **%** | **95% CI** | |
| **All causes** | 16.2 | 14.0 | 18.4 | 77.8 | 69.7 | 88.4 | 6.7 | 6.0 | 7.6 |
| **Pneumonia** | 104.9 | 91.7 | 117.6 | 99.1 | 93.9 | 106.1 | 2.9 | 2.8 | 3.1 |
| **Other respiratory diseases** | 37.9 | 33.8 | 42.5 | 108.3 | 99.9 | 117.9 | 3.6 | 3.3 | 3.9 |
| **Diabetes** | 32.2 | 25.5 | 38.6 | 61.8 | 53.9 | 73.9 | 6.1 | 5.3 | 7.3 |
| **Hypertensive heart disease** | 24.6 | 19.5 | 29.7 | 55.2 | 47.4 | 66.5 | 7.1 | 6.1 | 8.5 |
| **Other kidney disease** | 22.8 | 14.3 | 32.2 | 102.5 | 78.0 | 152.6 | 6.1 | 4.6 | 9.0 |
| **Homicide** | 19.1 | 11.3 | 27.2 | 0.2 | 0.1 | 0.3 | 0.3 | 0.2 | 0.5 |
| **Ill-defined** | 14.0 | 10.8 | 17.4 | 88.7 | 73.6 | 111.8 | 9.2 | 7.6 | 11.6 |
| **Alcohol-related causes** | 13.7 | 10.0 | 17.4 | 18.5 | 15.1 | 24.5 | 5.7 | 4.6 | 7.5 |
| **Parkinson's disease** | 13.7 | 5.2 | 23.0 | 73.0 | 47.0 | 159.3 | 12.0 | 7.7 | 26.2 |
| **Alzheimer's and other dementias** | 12.8 | -1.1 | 27.4 | 93.9 | -414.7 | 508.4 | 16.6 | -73.3 | 89.9 |
| **Other cardiovascular diseases** | 11.8 | 4.9 | 18.5 | 54.6 | 36.8 | 123.9 | 11.5 | 7.8 | 26.1 |
| **Other heart disease** | 11.8 | 8.5 | 15.1 | 80.9 | 65.3 | 108.9 | 9.6 | 7.7 | 12.9 |
| **Stroke** | 11.4 | 4.7 | 18.3 | 59.3 | 39.1 | 135.6 | 12.9 | 8.5 | 29.5 |
| **IHD** | 11.1 | 4.8 | 18.0 | 60.0 | 39.5 | 130.4 | 12.5 | 8.3 | 27.2 |
| **Sepsis** | 9.2 | 3.5 | 15.0 | 186.9 | 120.8 | 460.7 | 10.0 | 6.5 | 24.6 |
| **Drug use disorders** | 8.2 | 1.9 | 14.0 | 5.4 | 3.3 | 19.4 | 4.9 | 3.0 | 17.8 |
| **Chronic kidney disease** | 7.6 | 0.1 | 15.8 | 149.6 | 67.2 | 869.7 | 17.8 | 8.0 | 103.3 |
| **Transport accidents** | 4.0 | 1.1 | 6.7 | 3.4 | 2.1 | 11.9 | 4.7 | 2.9 | 16.2 |
| **Chronic respiratory disease** | 3.9 | -3.3 | 11.1 | 164.2 | -2351.0 | 2599.0 | 22.7 | -324.5 | 358.8 |
| **All cancers** | 2.0 | -0.6 | 4.9 | 101.8 | -820.2 | 1182.8 | 28.0 | -225.8 | 325.7 |
| **Falls** | 1.0 | -7.7 | 10.8 | 23.8 | -662.8 | 569.2 | 23.8 | -663.0 | 569.4 |
| **Lung cancer** | -3.3 | -8.2 | 2.2 | -37.4 | -501.9 | 345.9 | -12.8 | -172.2 | 118.7 |
| **Suicide** | -6.7 | -9.0 | -4.6 | -0.1 | -0.2 | -0.1 | -1.5 | -2.2 | -1.1 |
| **Influenza** | -61.2 | -75.2 | -45.7 | -7.8 | -14.4 | -4.1 | -1.6 | -2.9 | -0.8 |

95% CI: 95% confidence interval.

**Table 4** Excess mortality and ratio of COVID-19 (UC and CC) to excess mortality, females, US, 2020

|  | **Excess mortality** | | | **Ratio of COVID-19 UC to excess mortality** | | | **Ratio of COVID-19 CC to excess mortality** | | |
| --- | --- | --- | --- | --- | --- | --- | --- | --- | --- |
| **Causes** | **%** | **95% CI** | | **%** | **95% CI** | | **%** | **95% CI** | |
| **All causes** | 13.9 | 11.6 | 16.3 | 78.9 | 68.6 | 92.3 | 8.7 | 7.5 | 10.1 |
| **Pneumonia** | 81.2 | 68.0 | 93.7 | 104.8 | 96.9 | 116.1 | 3.3 | 3.1 | 3.7 |
| **Other respiratory diseases** | 27.7 | 23.7 | 32.3 | 116.1 | 103.2 | 131.3 | 4.6 | 4.1 | 5.2 |
| **Diabetes** | 33.3 | 26.4 | 40.5 | 55.8 | 48.4 | 66.7 | 6.2 | 5.4 | 7.4 |
| **Hypertensive heart disease** | 24.4 | 17.3 | 31.5 | 49.1 | 40.2 | 65.6 | 7.7 | 6.3 | 10.3 |
| **Other kidney disease** | 18.9 | 9.2 | 27.5 | 84.5 | 62.3 | 159.2 | 7.8 | 5.7 | 14.7 |
| **Homicide** | 8.0 | 1.1 | 15.4 | 0.0 | 0.0 | 0.0 | 1.0 | 0.5 | 4.3 |
| **Ill-defined** | 15.2 | 11.3 | 19.1 | 67.0 | 55.2 | 87.4 | 9.8 | 8.1 | 12.8 |
| **Alcohol-related causes** | 12.3 | 8.1 | 16.9 | 22.1 | 16.7 | 32.3 | 6.4 | 4.8 | 9.4 |
| **Parkinson's disease** | 14.6 | 3.8 | 26.4 | 62.6 | 38.5 | 216.0 | 13.6 | 8.4 | 47.0 |
| **Alzheimer's and other dementias** | 13.5 | 0.2 | 28.3 | 75.8 | 34.2 | 454.6 | 16.9 | 7.6 | 101.2 |
| **Other cardiovascular diseases** | 12.0 | 4.0 | 20.1 | 43.6 | 27.7 | 119.4 | 10.3 | 6.6 | 28.3 |
| **Other heart disease** | 9.7 | 5.9 | 14.2 | 82.4 | 58.5 | 131.9 | 12.3 | 8.7 | 19.6 |
| **Stroke** | 7.5 | -1.4 | 16.5 | 61.9 | -153.4 | 555.0 | 17.5 | -43.3 | 156.8 |
| **IHD** | 11.1 | 2.9 | 19.7 | 51.1 | 31.0 | 166.7 | 14.1 | 8.5 | 45.9 |
| **Sepsis** | 5.8 | 0.5 | 11.6 | 205.3 | 103.8 | 1157.9 | 15.1 | 7.7 | 85.4 |
| **Drug use disorders** | 6.4 | 0.9 | 12.5 | 7.1 | 3.7 | 34.0 | 5.2 | 2.7 | 24.8 |
| **Chronic kidney disease** | 5.3 | -2.0 | 12.8 | 173.9 | -960.4 | 1508.4 | 24.2 | -133.7 | 209.9 |
| **Transport accidents** | -0.2 | -3.7 | 3.5 | -3.0 | -96.7 | 70.1 | -3.0 | -97.4 | 70.7 |
| **Chronic respiratory disease** | 1.2 | -6.2 | 8.3 | 145.2 | -3120.1 | 2778.3 | 22.0 | -473.7 | 421.8 |
| **All cancers** | 0.8 | -1.6 | 3.4 | 100.7 | -1364.5 | 2461.1 | 33.3 | -451.2 | 813.9 |
| **Falls** | -2.8 | -13.7 | 10.4 | -18.7 | -407.5 | 493.3 | -20.2 | -441.3 | 534.3 |
| **Lung cancer** | -4.8 | -9.5 | -0.1 | -28.2 | -221.3 | -11.1 | -10.4 | -81.6 | -4.1 |
| **Suicide** | -17.9 | -21.3 | -14.4 | -0.1 | -0.1 | -0.1 | -0.7 | -0.8 | -0.5 |
| **Influenza** | -66.0 | -80.0 | -51.4 | -4.1 | -7.5 | -2.0 | -0.7 | -1.4 | -0.4 |

95% CI: 95% confidence interval.

**Table 5** Excess mortality and ratio of COVID-19 (UC and CC) to excess mortality, males, US, 2021

|  | **Excess mortality** | | | **Ratio of COVID-19 UC to excess mortality** | | | **Ratio of COVID-19 CC to excess mortality** | | |
| --- | --- | --- | --- | --- | --- | --- | --- | --- | --- |
| **Causes** | **%** | **95% CI** | | **%** | **95% CI** | | **%** | **95% CI** | |
| **All causes** | 21.5 | 19.3 | 23.7 | 71.5 | 66.1 | 78.4 | 7.1 | 6.5 | 7.8 |
| **Pneumonia** | 149.3 | 134.2 | 166.7 | 102.7 | 98.3 | 107.4 | 3.9 | 3.8 | 4.1 |
| **Other kidney disease** | 67.9 | 56.1 | 80.6 | 62.7 | 56.8 | 70.4 | 4.0 | 3.7 | 4.5 |
| **Other respiratory diseases** | 52.3 | 47.6 | 56.9 | 102.5 | 97.1 | 109.1 | 3.9 | 3.7 | 4.2 |
| **Diabetes** | 35.9 | 29.6 | 42.7 | 57.0 | 50.3 | 65.9 | 7.4 | 6.6 | 8.6 |
| **Hypertensive heart disease** | 27.2 | 22.0 | 32.5 | 45.8 | 39.9 | 54.4 | 8.4 | 7.3 | 9.9 |
| **Homicide** | 26.7 | 19.3 | 35.4 | 0.1 | 0.1 | 0.1 | 0.4 | 0.3 | 0.5 |
| **Ill-defined** | 22.4 | 18.9 | 26.0 | 72.8 | 64.6 | 83.8 | 8.6 | 7.6 | 9.9 |
| **Alcohol-related causes** | 22.1 | 18.2 | 26.0 | 15.8 | 13.8 | 18.7 | 5.4 | 4.7 | 6.3 |
| **Other cardiovascular diseases** | 19.6 | 12.5 | 27.3 | 47.3 | 36.1 | 69.7 | 10.8 | 8.3 | 15.9 |
| **Sepsis** | 17.9 | 12.1 | 25.0 | 130.7 | 99.4 | 183.8 | 8.1 | 6.2 | 11.4 |
| **Drug use disorders** | 17.9 | 11.5 | 24.4 | 5.5 | 4.3 | 8.1 | 5.4 | 4.2 | 7.9 |
| **Other heart disease** | 16.4 | 13.2 | 19.5 | 72.7 | 62.9 | 87.8 | 10.8 | 9.3 | 13.0 |
| **Transport accidents** | 15.8 | 12.9 | 19.0 | 1.0 | 0.9 | 1.2 | 1.7 | 1.4 | 2.0 |
| **Stroke** | 14.2 | 7.1 | 21.5 | 48.3 | 33.9 | 91.0 | 15.0 | 10.5 | 28.3 |
| **IHD** | 14.0 | 7.8 | 20.3 | 49.3 | 35.8 | 83.4 | 14.1 | 10.2 | 23.8 |
| **Falls** | 5.3 | -3.7 | 14.6 | 21.9 | -212.2 | 209.8 | 32.0 | -310.6 | 307.1 |
| **All cancers** | 5.2 | 2.5 | 8.0 | 53.5 | 35.3 | 109.6 | 20.7 | 13.6 | 42.3 |
| **Chronic kidney disease** | 4.1 | -3.7 | 11.5 | 202.7 | -2119.5 | 2464.9 | 33.2 | -347.4 | 404.0 |
| **Parkinson's disease** | 3.9 | -4.1 | 12.7 | 104.8 | -1173.4 | 1229.4 | 34.6 | -387.3 | 405.7 |
| **Chronic respiratory disease** | 2.2 | -4.8 | 9.7 | 178.1 | -2271.4 | 3077.5 | 35.1 | -447.3 | 606.0 |
| **Alzheimer's and other dementias** | 2.1 | -10.1 | 16.3 | 74.2 | -1383.8 | 1904.2 | 23.4 | -436.8 | 601.1 |
| **Lung cancer** | -0.1 | -5.6 | 5.2 | -28.5 | -1014.1 | 998.8 | -14.4 | -512.1 | 504.4 |
| **Suicide** | -1.9 | -4.1 | 0.6 | -0.8 | -9.4 | 4.1 | -9.1 | -107.4 | 46.5 |
| **Influenza** | -95.4 | -97.2 | -93.2 | -1.1 | -1.6 | -0.6 | -0.1 | -0.2 | -0.1 |

95% CI: 95% confidence interval.

**Table 6** Excess mortality and ratio of COVID-19 (UC and CC) to excess mortality, females, US, 2021

|  | **Excess mortality** | | | | **Ratio of COVID-19 UC to excess mortality** | | | **Ratio of COVID-19 CC to excess mortality** | | |
| --- | --- | --- | --- | --- | --- | --- | --- | --- | --- | --- |
| **Causes** | **%** | **95% CI** | | | **%** | **95% CI** | | **%** | **95% CI** | |
| **All causes** | 21.8 | 19.3 | 24.1 | 62.1 | | 57.3 | 68.8 | 7.7 | 7.1 | 8.5 |
| **Pneumonia** | 125.3 | 109.3 | 143.1 | 106.3 | | 100.4 | 113.2 | 4.7 | 4.4 | 5.0 |
| **Other kidney disease** | 63.8 | 51.5 | 77.8 | 50.0 | | 44.5 | 57.4 | 4.3 | 3.8 | 4.9 |
| **Other respiratory diseases** | 43.3 | 38.4 | 48.1 | 101.5 | | 94.4 | 110.7 | 4.9 | 4.5 | 5.3 |
| **Diabetes** | 43.2 | 36.0 | 51.7 | 48.0 | | 42.5 | 54.7 | 7.1 | 6.3 | 8.1 |
| **Hypertensive heart disease** | 33.3 | 25.2 | 40.9 | 34.5 | | 29.6 | 42.9 | 8.0 | 6.9 | 10.0 |
| **Homicide** | 15.5 | 8.1 | 23.7 | 0.3 | | 0.2 | 0.5 | 0.6 | 0.4 | 1.2 |
| **Ill-defined** | 27.0 | 22.2 | 31.9 | 49.9 | | 43.8 | 58.3 | 8.1 | 7.1 | 9.5 |
| **Alcohol-related causes** | 19.9 | 14.8 | 24.6 | 19.4 | | 16.3 | 25.0 | 6.9 | 5.8 | 8.9 |
| **Other cardiovascular diseases** | 23.9 | 14.1 | 34.2 | 31.3 | | 23.6 | 48.6 | 9.2 | 7.0 | 14.4 |
| **Sepsis** | 16.4 | 10.3 | 22.6 | 111.4 | | 84.6 | 168.4 | 9.2 | 7.0 | 13.9 |
| **Drug use disorders** | 17.2 | 11.4 | 23.3 | 6.3 | | 4.8 | 9.0 | 5.7 | 4.4 | 8.2 |
| **Other heart disease** | 19.0 | 14.2 | 23.5 | 53.7 | | 44.9 | 68.8 | 9.6 | 8.0 | 12.3 |
| **Transport accidents** | 12.9 | 9.0 | 16.9 | 0.9 | | 0.7 | 1.3 | 2.6 | 2.1 | 3.7 |
| **Stroke** | 16.2 | 7.1 | 25.7 | 29.8 | | 20.5 | 63.1 | 12.7 | 8.7 | 26.9 |
| **IHD** | 18.7 | 9.9 | 27.1 | 30.6 | | 22.5 | 53.4 | 11.4 | 8.4 | 19.9 |
| **Falls** | 7.6 | -5.2 | 22.0 | 15.5 | | -155.3 | 199.0 | 24.0 | -239.9 | 307.4 |
| **All cancers** | 6.3 | 3.8 | 9.3 | 35.2 | | 24.7 | 57.7 | 16.3 | 11.4 | 26.7 |
| **Chronic kidney disease** | 6.7 | -1.1 | 14.2 | 129.2 | | -533.5 | 898.0 | 27.0 | -111.3 | 187.4 |
| **Parkinson's disease** | 9.8 | -1.1 | 21.5 | 49.0 | | -169.3 | 323.6 | 19.3 | -66.7 | 127.6 |
| **Chronic respiratory disease** | 2.4 | -4.4 | 9.8 | 162.4 | | -3125.4 | 2262.3 | 35.9 | -691.5 | 500.5 |
| **Alzheimer's and other dementias** | 10.1 | -2.5 | 24.8 | 51.2 | | -449.8 | 371.4 | 21.4 | -187.6 | 154.9 |
| **Lung cancer** | 0.0 | -5.0 | 5.3 | 25.3 | | -907.3 | 774.9 | 13.5 | -485.0 | 414.2 |
| **Suicide** | -15.5 | -19.1 | -11.8 | 0.0 | | -0.1 | 0.0 | -1.2 | -1.7 | -1.0 |
| **Influenza** | -95.9 | -97.8 | -93.5 | -0.7 | | -1.0 | -0.4 | -0.2 | -0.2 | -0.1 |

95% CI: 95% confidence interval.

**Table 7** Number of Expected, Observed, and Excess Deaths by Cause, Including COVID-19 UC and CC, by sex, 2020 and 2021

| **Causes** | **Year** | **Sex** | **Expected** | **Observed** | **Excess deaths** | **COVID UC** | **COVID CC** |
| --- | --- | --- | --- | --- | --- | --- | --- |
| **All cause** | 2020 | Male | 1,523,000 | 1,769,805 | 246,805 | 192,509 | 16,205 |
| **All cause** | 2021 | Male | 1,512,289 | 1,838,038 | 325,749 | 236,607 | 22,896 |
| **All cause** | 2020 | Female | 1,432,085 | 1,613,808 | 181,723 | 158,318 | 17,499 |
| **All cause** | 2021 | Female | 1,335,631 | 1,626,100 | 290,469 | 180,283 | 22,404 |
| **All cancers** | 2020 | Male | 362,353 | 370,745 | 8,392 | 8,031 | 2,226 |
| **All cancers** | 2021 | Male | 362,231 | 374,837 | 12,606 | 9,832 | 3,746 |
| **All cancers** | 2020 | Female | 326,222 | 324,091 | -2,131 | 5,669 | 1,847 |
| **All cancers** | 2021 | Female | 312,685 | 328,176 | 15,491 | 6,928 | 3,179 |
| **Alcohol-related causes** | 2020 | Male | 88,487 | 100,996 | 12,509 | 2,333 | 691 |
| **Alcohol-related causes** | 2021 | Male | 90,327 | 110,292 | 19,965 | 3,252 | 1,080 |
| **Alcohol-related causes** | 2020 | Female | 40,978 | 45,918 | 4,940 | 1,194 | 327 |
| **Alcohol-related causes** | 2021 | Female | 40,124 | 50,305 | 10,181 | 1,706 | 585 |
| **Chronic kidney disease** | 2020 | Male | 136,607 | 144,079 | 7,472 | 15,802 | 1,853 |
| **Chronic kidney disease** | 2021 | Male | 142,801 | 146,643 | 3,843 | 15,365 | 2,501 |
| **Chronic kidney disease** | 2020 | Female | 120,281 | 127,783 | 7,502 | 12,130 | 1,713 |
| **Chronic kidney disease** | 2021 | Female | 126,933 | 129,898 | 2,965 | 11,123 | 2,320 |
| **Chronic respiratory disease** | 2020 | Male | 171,133 | 176,639 | 5,506 | 14,486 | 1,972 |
| **Chronic respiratory disease** | 2021 | Male | 158,427 | 172,638 | 14,211 | 15,053 | 2,898 |
| **Chronic respiratory disease** | 2020 | Female | 157,778 | 166,127 | 8,350 | 12,758 | 1,946 |
| **Chronic respiratory disease** | 2021 | Female | 153,873 | 161,666 | 7,793 | 12,626 | 2,778 |
| **Dementias** | 2020 | Male | 167,646 | 180,002 | 12,356 | 20,185 | 3,572 |
| **Dementias** | 2021 | Male | 151,209 | 161,449 | 10,240 | 10,751 | 3,421 |
| **Dementias** | 2020 | Female | 276,212 | 320,070 | 43,858 | 29,529 | 6,589 |
| **Dementias** | 2021 | Female | 277,448 | 283,067 | 5,620 | 14,823 | 6,190 |
| **Diabetes** | 2020 | Male | 169,109 | 218,033 | 48,924 | 32,973 | 3,201 |
| **Diabetes** | 2021 | Male | 170,644 | 225,425 | 54,781 | 34,286 | 4,426 |
| **Diabetes** | 2020 | Female | 137,641 | 170,027 | 32,386 | 23,637 | 2,649 |
| **Diabetes** | 2021 | Female | 126,930 | 173,976 | 47,046 | 24,634 | 3,693 |
| **Drug use disorders** | 2020 | Male | 68,615 | 75,909 | 7,294 | 317 | 285 |
| **Drug use disorders** | 2021 | Male | 77,338 | 88,516 | 11,178 | 758 | 723 |
| **Drug use disorders** | 2020 | Female | 30,182 | 32,340 | 2,158 | 155 | 104 |
| **Drug use disorders** | 2021 | Female | 32,249 | 37,630 | 5,381 | 365 | 320 |
| **Falls** | 2020 | Male | 26,525 | 26,711 | 186 | 469 | 458 |
| **Falls** | 2021 | Male | 28,215 | 28,236 | 21 | 403 | 591 |
| **Falls** | 2020 | Female | 26,552 | 27,108 | 556 | 494 | 531 |
| **Falls** | 2021 | Female | 27,604 | 28,386 | 783 | 398 | 607 |
| **Homicide** | 2020 | Male | 16,429 | 19,878 | 3,449 | 5 | 9 |
| **Homicide** | 2021 | Male | 16,255 | 20,959 | 4,704 | 4 | 19 |
| **Homicide** | 2020 | Female | 4,260 | 4,643 | 383 | - | 4 |
| **Homicide** | 2021 | Female | 4,430 | 4,968 | 538 | 2 | 5 |
| **Hypertensive heart disease** | 2020 | Male | 270,332 | 342,890 | 72,558 | 37,590 | 4,693 |
| **Hypertensive heart disease** | 2021 | Male | 291,366 | 357,213 | 65,847 | 35,736 | 6,334 |
| **Hypertensive heart disease** | 2020 | Female | 254,933 | 328,544 | 73,612 | 31,770 | 5,017 |
| **Hypertensive heart disease** | 2021 | Female | 257,824 | 333,822 | 75,998 | 28,612 | 6,656 |
| **Ill-defined** | 2020 | Male | 241,233 | 279,943 | 38,710 | 30,938 | 3,098 |
| **Ill-defined** | 2021 | Male | 238,436 | 301,797 | 63,361 | 41,136 | 4,695 |
| **Ill-defined** | 2020 | Female | 251,610 | 296,131 | 44,521 | 26,422 | 3,941 |
| **Ill-defined** | 2021 | Female | 238,928 | 305,653 | 66,725 | 32,145 | 5,297 |
| **IHD** | 2020 | Male | 340,533 | 370,553 | 30,020 | 22,143 | 4,578 |
| **IHD** | 2021 | Male | 309,096 | 372,931 | 63,835 | 22,944 | 6,392 |
| **IHD** | 2020 | Female | 238,783 | 246,687 | 7,905 | 12,871 | 3,555 |
| **IHD** | 2021 | Female | 207,300 | 239,900 | 32,600 | 11,623 | 4,252 |
| **Influenza** | 2020 | Male | 18,646 | 4,525 | -14,121 | 585 | 122 |
| **Influenza** | 2021 | Male | 8,788 | 553 | -8,235 | 132 | 17 |
| **Influenza** | 2020 | Female | 9,985 | 4,236 | -5,749 | 369 | 70 |
| **Influenza** | 2021 | Female | 16,510 | 536 | -15,974 | 101 | 22 |
| **Lung cancer** | 2020 | Male | 79,774 | 80,701 | 927 | 1,281 | 447 |
| **Lung cancer** | 2021 | Male | 81,882 | 79,735 | -2,147 | 1,508 | 760 |
| **Lung cancer** | 2020 | Female | 71,696 | 69,296 | -2,400 | 1,023 | 373 |
| **Lung cancer** | 2021 | Female | 68,022 | 69,423 | 1,401 | 1,230 | 642 |
| **Other cardiovascular diseases** | 2020 | Male | 71,816 | 83,950 | 12,134 | 4,875 | 1,006 |
| **Other cardiovascular diseases** | 2021 | Male | 70,760 | 88,668 | 17,908 | 7,020 | 1,553 |
| **Other cardiovascular diseases** | 2020 | Female | 66,590 | 75,336 | 8,746 | 3,570 | 851 |
| **Other cardiovascular diseases** | 2021 | Female | 60,541 | 77,308 | 16,767 | 4,603 | 1,360 |
| **Other heart disease** | 2020 | Male | 505,319 | 572,492 | 67,173 | 49,220 | 5,722 |
| **Other heart disease** | 2021 | Male | 513,879 | 596,965 | 83,086 | 62,473 | 8,998 |
| **Other heart disease** | 2020 | Female | 506,101 | 533,471 | 27,370 | 39,121 | 5,886 |
| **Other heart disease** | 2021 | Female | 451,655 | 542,427 | 90,772 | 45,967 | 8,249 |
| **Other kidney disease** | 2020 | Male | 56,971 | 73,522 | 16,551 | 14,344 | 821 |
| **Other kidney disease** | 2021 | Male | 54,622 | 95,257 | 40,635 | 24,633 | 1,563 |
| **Other kidney disease** | 2020 | Female | 50,838 | 58,789 | 7,951 | 7,881 | 738 |
| **Other kidney disease** | 2021 | Female | 43,853 | 73,155 | 29,302 | 14,058 | 1,209 |
| **Other respiratory diseases** | 2020 | Male | 242,133 | 333,860 | 91,727 | 99,835 | 3,266 |
| **Other respiratory diseases** | 2021 | Male | 232,133 | 374,221 | 142,088 | 132,949 | 5,069 |
| **Other respiratory diseases** | 2020 | Female | 227,360 | 296,977 | 69,617 | 75,392 | 2,992 |
| **Other respiratory diseases** | 2021 | Female | 225,912 | 322,158 | 96,246 | 97,522 | 4,643 |
| **Parkinson's disease** | 2020 | Male | 33,863 | 39,013 | 5,150 | 3,479 | 567 |
| **Parkinson's disease** | 2021 | Male | 34,374 | 36,419 | 2,045 | 2,078 | 684 |
| **Parkinson's disease** | 2020 | Female | 20,890 | 24,505 | 3,615 | 1,966 | 431 |
| **Parkinson's disease** | 2021 | Female | 19,377 | 22,483 | 3,106 | 1,038 | 404 |
| **Pneumonia** | 2020 | Male | 95,452 | 196,252 | 100,800 | 100,135 | 2,905 |
| **Pneumonia** | 2021 | Male | 90,232 | 232,216 | 141,984 | 143,741 | 5,471 |
| **Pneumonia** | 2020 | Female | 89,119 | 157,604 | 68,485 | 74,358 | 2,335 |
| **Pneumonia** | 2021 | Female | 78,150 | 179,194 | 101,044 | 105,286 | 4,615 |
| **Sepsis** | 2020 | Male | 112,421 | 125,301 | 12,880 | 20,136 | 1,052 |
| **Sepsis** | 2021 | Male | 114,046 | 136,626 | 22,580 | 27,570 | 1,687 |
| **Sepsis** | 2020 | Female | 108,100 | 116,787 | 8,687 | 13,507 | 1,004 |
| **Sepsis** | 2021 | Female | 113,794 | 124,336 | 10,542 | 19,093 | 1,580 |
| **Stroke** | 2020 | Male | 124,407 | 135,304 | 10,897 | 8,423 | 1,808 |
| **Stroke** | 2021 | Male | 121,940 | 138,285 | 16,345 | 8,617 | 2,596 |
| **Stroke** | 2020 | Female | 150,882 | 159,073 | 8,191 | 7,144 | 2,024 |
| **Stroke** | 2021 | Female | 136,772 | 160,500 | 23,728 | 6,669 | 2,844 |
| **Suicide** | 2020 | Male | 39,188 | 36,585 | -2,603 | 4 | 40 |
| **Suicide** | 2021 | Male | 39,299 | 38,392 | -907 | 6 | 74 |
| **Suicide** | 2020 | Female | 11,225 | 9,446 | -1,779 | 2 | 14 |
| **Suicide** | 2021 | Female | 12,048 | 9,850 | -2,198 | 1 | 23 |
| **Transport accidents** | 2020 | Male | 31,333 | 32,881 | 1,548 | 46 | 61 |
| **Transport accidents** | 2021 | Male | 31,196 | 36,113 | 4,917 | 54 | 85 |
| **Transport accidents** | 2020 | Female | 12,362 | 12,258 | -104 | 15 | 16 |
| **Transport accidents** | 2021 | Female | 12,155 | 13,832 | 1,677 | 18 | 46 |

**Table 8** Excess mortality and ratio of COVID-19 (UC and CC) to excess mortality, hospital deaths, males, US, 2020-2021

|  | **Excess mortality** | | | **Ratio of COVID-19 UC to excess mortality** | | | **Ratio of COVID-19 CC to excess mortality** | | |
| --- | --- | --- | --- | --- | --- | --- | --- | --- | --- |
| **Causes** | **%** | **95% CI** | | **%** | **95% CI** | | **%** | **95% CI** | |
| **All causes** | 33.2 | 31.4 | 35.1 | 92.9 | 89.1 | 96.9 | 4.5 | 4.4 | 4.7 |
| **Pneumonia** | 158.8 | 147.9 | 172.3 | 100.4 | 97.4 | 103.4 | 2.5 | 2.4 | 2.5 |
| **Other kidney disease** | 62.8 | 55.5 | 70.3 | 75.1 | 70.1 | 81.2 | 3.7 | 3.4 | 4.0 |
| **Other respiratory diseases** | 62.7 | 58.9 | 66.6 | 106.0 | 102.2 | 110.3 | 2.7 | 2.6 | 2.9 |
| **Diabetes** | 57.4 | 51.8 | 63.2 | 81.7 | 76.9 | 87.6 | 5.1 | 4.8 | 5.5 |
| **Hypertensive heart disease** | 39.1 | 34.8 | 43.5 | 79.6 | 73.8 | 87.0 | 5.9 | 5.5 | 6.5 |
| **Alzheimer's and other dementias** | 27.0 | 16.9 | 36.9 | 105.1 | 83.1 | 154.9 | 9.1 | 7.2 | 13.5 |
| **Ill-defined** | 24.1 | 21.8 | 26.6 | 98.8 | 91.4 | 107.5 | 6.3 | 5.8 | 6.8 |
| **Other heart disease** | 21.6 | 19.2 | 24.1 | 94.1 | 86.1 | 103.8 | 7.1 | 6.5 | 7.8 |
| **Parkinson's disease** | 21.0 | 12.9 | 28.5 | 113.6 | 87.9 | 174.2 | 8.9 | 6.9 | 13.7 |
| **Homicide** | 20.5 | 13.5 | 28.0 | 0.2 | 0.1 | 0.3 | 0.8 | 0.6 | 1.1 |
| **Sepsis** | 18.6 | 14.1 | 22.7 | 129.2 | 109.4 | 164.3 | 6.4 | 5.4 | 8.2 |
| **Other cardiovascular diseases** | 17.4 | 12.6 | 22.5 | 79.3 | 63.9 | 104.9 | 10.2 | 8.2 | 13.5 |
| **Alcohol-related causes** | 17.1 | 13.5 | 20.6 | 36.2 | 30.8 | 44.2 | 8.4 | 7.1 | 10.2 |
| **Chronic kidney disease** | 16.0 | 11.0 | 21.3 | 144.8 | 114.0 | 202.3 | 11.4 | 9.0 | 15.9 |
| **Stroke** | 16.0 | 11.3 | 20.9 | 73.0 | 57.9 | 98.7 | 12.4 | 9.8 | 16.7 |
| **IHD** | 13.8 | 9.2 | 18.9 | 98.9 | 75.5 | 142.8 | 13.2 | 10.1 | 19.1 |
| **Chronic respiratory disease** | 11.8 | 6.3 | 17.9 | 172.0 | 120.0 | 308.9 | 14.1 | 9.9 | 25.4 |
| **Transport accidents** | 6.4 | 3.9 | 9.1 | 4.7 | 3.4 | 7.5 | 7.0 | 5.0 | 11.1 |
| **All cancers** | 2.7 | 0.1 | 5.3 | 246.7 | 109.6 | 1545.5 | 41.5 | 18.4 | 260.2 |
| **Falls** | 2.2 | -5.3 | 9.1 | 41.9 | -807.1 | 853.7 | 45.1 | -869.1 | 919.3 |
| **Drug use disorders** | 0.2 | -4.0 | 4.8 | 44.0 | -1540.3 | 1423.6 | 26.0 | -911.1 | 842.1 |
| **Lung cancer** | -1.9 | -6.3 | 2.4 | -154.8 | -2502.0 | 2782.2 | -32.6 | -527.2 | 586.2 |
| **Suicide** | -4.3 | -7.7 | -0.6 | -1.1 | -4.9 | -0.6 | -7.5 | -33.5 | -3.9 |
| **Influenza** | -80.2 | -85.7 | -73.7 | -1.6 | -2.3 | -1.1 | -0.2 | -0.3 | -0.1 |

95% CI: 95% confidence interval.

**Table 9** Excess mortality and ratio of COVID-19 (UC and CC) to excess mortality, hospital deaths, females, US, 2020-2021

|  | **Excess mortality** | | | **Ratio of COVID-19 UC to excess mortality** | | | | **Ratio of COVID-19 CC to excess mortality** | | |
| --- | --- | --- | --- | --- | --- | --- | --- | --- | --- | --- |
| **Causes** | **%** | **95% CI** | | **%** | **95% CI** | | **%** | | **95% CI** | |
| **All causes** | 29.8 | 27.9 | 31.7 | 90.7 | 86.6 | 95.5 | 4.9 | | 4.7 | 5.2 |
| **Pneumonia** | 126.9 | 116.1 | 138.2 | 104.5 | 100.9 | 108.8 | 2.9 | | 2.8 | 3.0 |
| **Other kidney disease** | 55.9 | 48.8 | 64.2 | 62.2 | 56.9 | 68.0 | 3.7 | | 3.4 | 4.1 |
| **Other respiratory diseases** | 48.5 | 45.0 | 52.1 | 110.8 | 105.8 | 116.8 | 3.4 | | 3.2 | 3.5 |
| **Diabetes** | 59.7 | 54.1 | 65.9 | 75.2 | 70.8 | 80.3 | 4.9 | | 4.6 | 5.2 |
| **Hypertensive heart disease** | 39.9 | 34.3 | 45.5 | 73.2 | 66.9 | 81.9 | 5.8 | | 5.3 | 6.5 |
| **Alzheimer's and other dementias** | 24.8 | 14.1 | 35.1 | 99.8 | 76.2 | 160.8 | 9.3 | | 7.1 | 15.0 |
| **Ill-defined** | 22.5 | 19.9 | 25.1 | 88.6 | 81.0 | 98.0 | 6.2 | | 5.7 | 6.9 |
| **Other heart disease** | 20.4 | 17.2 | 23.4 | 85.4 | 76.2 | 98.4 | 7.0 | | 6.2 | 8.0 |
| **Parkinson's disease** | 23.6 | 14.6 | 33.5 | 97.9 | 74.0 | 146.8 | 8.4 | | 6.4 | 12.6 |
| **Homicide** | 8.4 | -0.2 | 17.0 | 0.7 | -1.8 | 4.6 | 3.1 | | -8.4 | 21.2 |
| **Sepsis** | 14.3 | 10.2 | 18.7 | 123.6 | 98.4 | 167.7 | 8.0 | | 6.4 | 10.9 |
| **Other cardiovascular diseases** | 17.2 | 12.4 | 22.5 | 61.8 | 49.2 | 82.7 | 8.8 | | 7.0 | 11.8 |
| **Alcohol-related causes** | 17.2 | 13.0 | 21.5 | 36.2 | 29.8 | 46.5 | 8.1 | | 6.7 | 10.4 |
| **Chronic kidney disease** | 13.9 | 9.3 | 18.8 | 144.1 | 111.7 | 208.4 | 12.4 | | 9.6 | 18.0 |
| **Stroke** | 11.7 | 6.3 | 17.0 | 70.0 | 50.5 | 123.1 | 13.9 | | 10.0 | 24.4 |
| **IHD** | 14.0 | 8.0 | 19.8 | 79.6 | 58.9 | 131.8 | 12.4 | | 9.2 | 20.6 |
| **Chronic respiratory disease** | 8.4 | 3.6 | 13.6 | 211.6 | 136.0 | 473.4 | 17.8 | | 11.4 | 39.8 |
| **Transport accidents** | 1.3 | -2.1 | 4.7 | 10.0 | -149.5 | 153.4 | 15.1 | | -224.7 | 230.5 |
| **All cancers** | 2.3 | -0.2 | 5.0 | 227.2 | -942.2 | 1474.2 | 43.8 | | -181.5 | 284.0 |
| **Falls** | 2.8 | -5.7 | 10.9 | 35.8 | -527.4 | 461.7 | 38.2 | | -563.5 | 493.3 |
| **Drug use disorders** | -2.9 | -7.3 | 1.9 | -48.4 | -547.8 | 514.7 | -26.9 | | -304.3 | 285.9 |
| **Lung cancer** | -2.7 | -6.5 | 1.2 | -146.1 | -1463.8 | 1522.7 | -29.5 | | -295.1 | 307.0 |
| **Suicide** | -21.7 | -26.7 | -16.5 | -0.2 | -0.3 | -0.1 | -1.5 | | -2.1 | -1.2 |
| **Influenza** | -80.4 | -86.2 | -74.4 | -1.1 | -1.5 | -0.7 | -0.1 | | -0.2 | -0.1 |

95% CI: 95% confidence interval.

**Table 10** Excess mortality and ratio of COVID-19 (UC and CC) to excess mortality, home deaths, males, US, 2020-2021

|  | **Excess mortality** | | | **Ratio of COVID-19 UC to excess mortality** | | | **Ratio of COVID-19 CC to excess mortality** | | |
| --- | --- | --- | --- | --- | --- | --- | --- | --- | --- |
| **Causes** | **%** | **95% CI** | | **%** | **95% CI** | | **%** | **95% CI** | |
| **All causes** | 20.2 | 18.4 | 22.1 | 15.6 | 14.5 | 16.9 | 4.2 | 3.9 | 4.6 |
| **Pneumonia** | 65.3 | 53.9 | 76.6 | 83.0 | 75.4 | 93.9 | 10.9 | 9.9 | 12.3 |
| **Diabetes** | 27.6 | 23.3 | 31.9 | 17.7 | 15.8 | 20.3 | 5.5 | 4.9 | 6.3 |
| **Hypertensive heart disease** | 26.5 | 22.9 | 30.0 | 13.9 | 12.6 | 15.6 | 5.1 | 4.6 | 5.7 |
| **Other cardiovascular diseases** | 23.9 | 18.3 | 29.3 | 7.0 | 5.9 | 8.7 | 4.4 | 3.7 | 5.5 |
| **Alcohol-related causes** | 22.4 | 18.7 | 25.9 | 3.8 | 3.4 | 4.5 | 2.4 | 2.1 | 2.8 |
| **Alzheimer's and other dementias** | 22.3 | 14.0 | 31.6 | 11.6 | 8.8 | 17.3 | 5.7 | 4.3 | 8.5 |
| **Other respiratory diseases** | 22.0 | 15.8 | 28.4 | 48.7 | 39.8 | 64.8 | 6.2 | 5.1 | 8.3 |
| **Ill-defined** | 22.0 | 17.1 | 26.8 | 14.6 | 12.5 | 18.1 | 4.6 | 4.0 | 5.7 |
| **Other kidney disease** | 21.3 | 13.6 | 29.0 | 13.3 | 10.4 | 19.7 | 4.9 | 3.8 | 7.2 |
| **Stroke** | 20.2 | 12.3 | 27.9 | 8.5 | 6.5 | 13.1 | 5.4 | 4.1 | 8.3 |
| **IHD** | 18.2 | 14.3 | 22.3 | 9.7 | 8.2 | 12.0 | 6.1 | 5.1 | 7.5 |
| **Parkinson's disease** | 17.6 | 10.2 | 25.5 | 12.0 | 8.7 | 19.3 | 6.0 | 4.4 | 9.6 |
| **Falls** | 16.7 | 8.9 | 25.1 | 3.9 | 2.8 | 7.0 | 7.5 | 5.4 | 13.3 |
| **All cancers** | 15.1 | 11.4 | 19.2 | 3.5 | 2.8 | 4.5 | 3.5 | 2.9 | 4.5 |
| **Other heart disease** | 15.0 | 9.6 | 20.0 | 14.1 | 11.0 | 21.0 | 6.4 | 5.0 | 9.4 |
| **Drug use disorders** | 14.1 | 9.0 | 19.4 | 2.0 | 1.5 | 3.0 | 3.6 | 2.7 | 5.3 |
| **Lung cancer** | 9.8 | 5.8 | 14.1 | 3.4 | 2.5 | 5.6 | 5.1 | 3.7 | 8.3 |
| **Chronic kidney disease** | 9.4 | 3.9 | 15.2 | 24.9 | 16.2 | 57.1 | 10.5 | 6.9 | 24.2 |
| **Sepsis** | 9.3 | 1.8 | 17.5 | 42.3 | 23.5 | 180.9 | 14.5 | 8.1 | 61.9 |
| **Chronic respiratory disease** | 7.2 | 2.7 | 11.9 | 30.3 | 19.0 | 76.3 | 12.7 | 8.0 | 32.0 |
| **Homicide** | 4.7 | -1.7 | 11.1 | 0.8 | -6.0 | 7.5 | 1.0 | -7.5 | 9.3 |
| **Transport accidents** | 0.5 | -9.5 | 10.8 | 5.9 | -135.9 | 200.3 | 4.2 | -97.8 | 144.1 |
| **Suicide** | -5.4 | -7.6 | -3.4 | -0.2 | -0.4 | -0.2 | -2.3 | -3.7 | -1.6 |
| **Influenza** | -46.2 | -59.6 | -32.5 | -43.5 | -76.8 | -25.8 | -9.6 | -16.9 | -5.7 |

95% CI: 95% confidence interval.

**Table 11** Excess mortality and ratio of COVID-19 (UC and CC) to excess mortality, home deaths, females, US, 2020-2021

|  | **Excess mortality** | | | **Ratio of COVID-19 UC to excess mortality** | | | **Ratio of COVID-19 CC to excess mortality** | | |
| --- | --- | --- | --- | --- | --- | --- | --- | --- | --- |
| **Causes** | **%** | **95% CI** | | **%** | **95% CI** | | **%** | **95% CI** | |
| **All causes** | 21.4 | 19.1 | 23.6 | 12.5 | 11.6 | 13.8 | 3.9 | 3.6 | 4.3 |
| **Pneumonia** | 59.0 | 47.0 | 71.0 | 81.5 | 72.6 | 94.7 | 11.8 | 10.5 | 13.7 |
| **Diabetes** | 32.5 | 27.4 | 37.8 | 14.6 | 13.1 | 16.7 | 5.0 | 4.5 | 5.7 |
| **Hypertensive heart disease** | 33.8 | 28.6 | 38.9 | 9.6 | 8.6 | 10.9 | 4.1 | 3.7 | 4.7 |
| **Other cardiovascular diseases** | 28.9 | 22.3 | 37.0 | 5.1 | 4.2 | 6.3 | 4.1 | 3.4 | 5.1 |
| **Alcohol-related causes** | 20.3 | 16.2 | 24.6 | 3.5 | 3.0 | 4.2 | 2.9 | 2.5 | 3.6 |
| **Alzheimer's and other dementias** | 23.7 | 14.6 | 33.0 | 9.2 | 7.1 | 13.9 | 5.1 | 3.9 | 7.7 |
| **Other respiratory diseases** | 20.5 | 14.0 | 27.3 | 42.9 | 34.0 | 59.2 | 6.1 | 4.8 | 8.4 |
| **Ill-defined** | 26.1 | 19.4 | 32.1 | 10.4 | 8.9 | 13.3 | 3.8 | 3.2 | 4.8 |
| **Other kidney disease** | 26.4 | 18.8 | 34.1 | 8.8 | 7.2 | 11.7 | 5.0 | 4.1 | 6.7 |
| **Stroke** | 24.6 | 15.7 | 33.9 | 6.0 | 4.7 | 8.8 | 4.3 | 3.4 | 6.3 |
| **IHD** | 22.4 | 17.0 | 27.7 | 6.7 | 5.6 | 8.4 | 4.9 | 4.1 | 6.1 |
| **Parkinson's disease** | 17.0 | 8.3 | 25.6 | 11.1 | 7.9 | 21.0 | 5.9 | 4.2 | 11.2 |
| **Falls** | 17.1 | 6.5 | 29.2 | 4.2 | 2.7 | 9.9 | 7.8 | 5.0 | 18.4 |
| **All cancers** | 15.0 | 11.0 | 18.9 | 2.7 | 2.2 | 3.5 | 3.2 | 2.6 | 4.2 |
| **Other heart disease** | 17.3 | 10.4 | 23.9 | 11.1 | 8.5 | 17.3 | 5.2 | 4.0 | 8.1 |
| **Drug use disorders** | 14.7 | 8.8 | 20.4 | 2.1 | 1.6 | 3.4 | 3.7 | 2.8 | 5.8 |
| **Lung cancer** | 10.2 | 5.9 | 14.4 | 3.0 | 2.2 | 4.9 | 4.7 | 3.4 | 7.7 |
| **Chronic kidney disease** | 11.6 | 4.1 | 18.3 | 18.7 | 12.5 | 48.7 | 9.3 | 6.2 | 24.1 |
| **Sepsis** | 15.5 | 7.8 | 23.7 | 22.2 | 15.6 | 41.4 | 10.2 | 7.2 | 19.0 |
| **Chronic respiratory disease** | 7.8 | 2.8 | 12.5 | 24.1 | 15.6 | 62.9 | 11.6 | 7.5 | 30.3 |
| **Homicide** | -1.7 | -8.0 | 4.9 | -0.7 | -13.5 | 12.7 | 0.0 | 0.0 | 0.0 |
| **Transport accidents** | -7.1 | -21.0 | 7.3 | -3.9 | -60.6 | 53.5 | -13.4 | -208.8 | 184.3 |
| **Suicide** | -19.4 | -22.6 | -15.9 | 0.0 | 0.0 | 0.0 | -0.6 | -0.8 | -0.5 |
| **Influenza** | -62.5 | -74.1 | -50.4 | -17.5 | -27.8 | -10.3 | -4.0 | -6.4 | -2.4 |

95% CI: 95% confidence interval.
